# Cytoglobin protects cancer cells from apoptosis by regulation of mitochondrial cardiolipin

**DOI:** 10.1038/s41598-020-79830-w

**Published:** 2021-01-13

**Authors:** Lorna S. Thorne, Garret Rochford, Timothy D. Williams, Andrew D. Southam, Giovanny Rodriguez-Blanco, Warwick B. Dunn, Nikolas J. Hodges

**Affiliations:** 1 School of Biosciences, University of Birmingham, Edgbaston, Birmingham, B15 2TT UK; 2 Phenome Centre Birmingham, University of Birmingham, Edgbaston, Birmingham, B15 2TT UK; 3 Institute of Metabolism and Systems Research, University of Birmingham, Edgbaston, Birmingham, B15 2TT UK

**Keywords:** Biochemistry, Cancer, Cell biology

## Abstract

Cytoglobin is important in the progression of oral squamous cell carcinoma but the molecular and cellular basis remain to be elucidated. In the current study, we develop a new cell model to study the function of cytoglobin in oral squamous carcinoma and response to cisplatin. Transcriptomic profiling showed cytoglobin mediated changes in expression of genes related to stress response, redox metabolism, mitochondrial function, cell adhesion, and fatty acid metabolism. Cellular and biochemical studies show that cytoglobin expression results in changes to phenotype associated with cancer progression including: increased cellular proliferation, motility and cell cycle progression. Cytoglobin also protects cells from cisplatin-induced apoptosis and oxidative stress with levels of the antioxidant glutathione increased and total and mitochondrial reactive oxygen species levels reduced. The mechanism of cisplatin resistance involved inhibition of caspase 9 activation and cytoglobin protected mitochondria from oxidative stress-induced fission. To understand the mechanism behind these phenotypic changes we employed lipidomic analysis and demonstrate that levels of the redox sensitive and apoptosis regulating cardiolipin are significantly up-regulated in cells expressing cytoglobin. In conclusion, our data shows that cytoglobin expression results in important phenotypic changes that could be exploited by cancer cells in vivo to facilitate disease progression.

## Introduction

Cytoglobin is a hexacoordinated heme containing globular proteins which is frequently silenced by promoter hypermethylation in head and neck, lung, ovarian, and melanoma cancers^1–5^. However, increases in cytoglobin expression in cancer are also reported^6^, supporting a complex role for cytoglobin in cancer progression. In vitro studies have identified candidate cellular functions of cytoglobin including: oxygen sensing, detoxification of reactive oxygen species (ROS) and protection against hypoxia^5,7,8^. Cytoglobin also promotes cell survival during oxidative stress induced by multiple stressors^2,8–12^. Although the mechanism is not yet fully elucidated, it may involve enzymatic detoxification of oxidants, because cytoglobin is reported to have peroxidase, superoxide dismutase, and nitric oxide dioxygenase activities^13–20^*.* Furthermore, cytoglobin also reduces cellular levels of lipid peroxides^21^. In head and neck squamous cell carcinoma (HNSCC) cytoglobin is linked with oncogenic phenotypes^22,23^. Cytoglobin is also an important determinant of cellular response to chemotherapeutic agents and radiotherapy. For example, knockdown in human glioma cells increases sensitivity to radiation-induced apoptosis^24^. Cytoglobin also reduces the sensitivity of murine myoblasts to etoposide-induced apoptosis^25^, and protects osteosarcoma cells from doxorubicin by inhibiting ubiquitin-mediated degradation of p53^26^. The generation of oxidative stress is often an important precursor to intrinsic apoptosis. Although agents such as 5-fluorouracil are employed, cisplatin and related compounds (carboplatin, oxaliplatin) remains the most widely used chemotherapeutic in the treatment of HNSCC^27,28^. Because many patients with locally advanced HNSCC receive radiotherapy combined with platinum-based chemotherapy (i.e. cisplatin), there is a clear need to better understand mechanisms of resistance to improve patient outcome because the 5-year survival rate is currently less than 40%. As well as DNA-crosslinking, cisplatin activity is known to involve mitochondrial ROS generation^29,30^, changes in mitochondrial membrane potential and damage to the electron transport chain triggering apoptosis and cell death^31–34^. Cytoglobin has been previously described as having peroxidase activity^14,21,35^, of which the local actions are uncertain within the overall context of the cellular redox state. Therefore, a better understanding of the mechanism by which cytoglobin protects cells from oxidative stress will result in novel targets for enhancing sensitivity and apoptosis signalling in cells to increase the effectiveness of cisplatin and other drugs in order to improve the outcome of existing treatments for cancers, including HNSCC. To study the role of cytoglobin in protection of cancer cells from cisplatin, we generated a novel oral squamous epithelial cell carcinoma cell model expressing cytoglobin and herein report that cytoglobin affords resistance to cisplatin through reduced levels of ROS and activation of caspase 9. Transcriptional and metabolomic studies identified changes in genes and metabolites regulating mitochondrial redox activity and levels of the lipid cardiolipin, which is known to alter the rates of apoptosis through changes in its affinity for cytochrome *c*, and subsequently effecting its release from the mitochondria.

In summary, our findings extend understanding of the fundamental cellular function of cytoglobin and identify a role in cisplatin resistance. Our findings show that targeting cytoglobin could be a new strategy to improve therapeutic response to cisplatin.

## Results

### Transcriptomic profiling of cytoglobin expressing cells

To study the effect of cytoglobin on phenotype we generated transgenic cells derived from the non-cytoglobin expressing PE/CA-PJ41 line^3^. Of the clones generated, two, LST421 and LST54, expressing high and intermediate levels of cytoglobin (Figure S1) were selected for further study. Biochemical analysis indicated that, as expected cytoglobin-expressing cells had elevated levels of the haem protein (Figure S2) but there were no differences in levels of either intracellular ATP or oxygen consumption (Figure S2). This suggests that in cytoglobin expressing cells, increased proliferation is not completely derived through increased oxygen consumption or increased overall ATP production.

**Figure 1:**
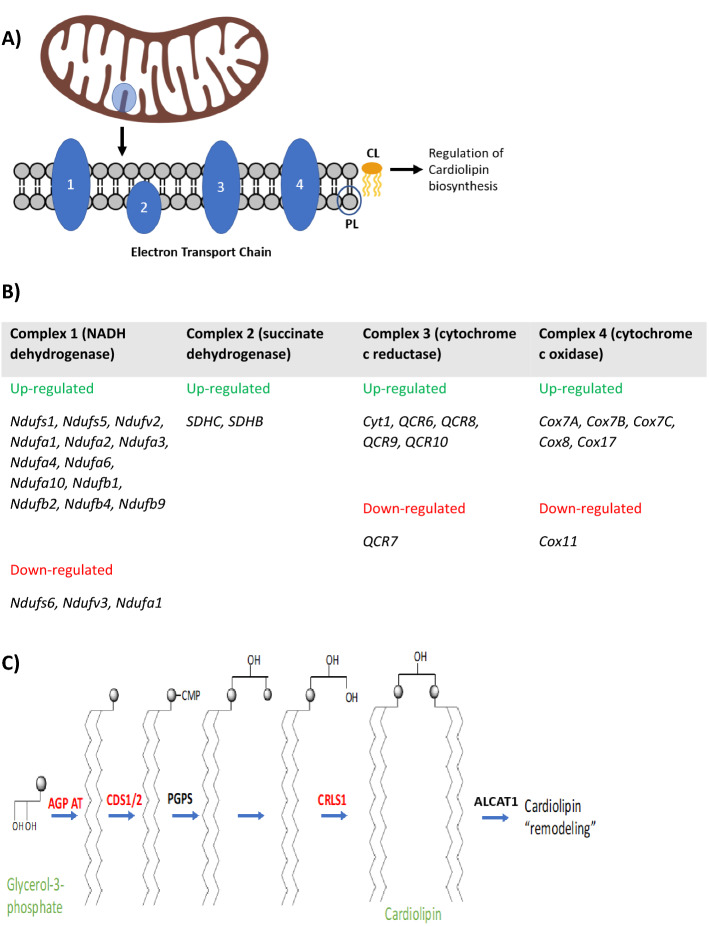
Genes related to mitochondrial electron transport chain (ETC) function and cardiolipin (CL) biosynthesis are significantly over-represented and differentially regulated in cytoglobin (LST421) expressing cells compared to non-cytoglobin expressing cells (NCE). (**A)** Multiple subunits of the electron transport chain complex were shown to be up-regulated: 13 in complex I, 2 in complex II, 5 in complex III and 4 in complex IV in cytoglobin expressing cells (highlighted in green). Downregulations in gene expression also occurred (highlighted in red): Complex I contained 3 genes, Complex III contained 1 and Complex IV contained 1 gene downregulated in cytoglobin expressing cells. (**B)** Biosynthesis of cardiolipin from glycerol 3-phosphate, 1-acylglycerol-3-phosphate O-acyl transferase 3 (AGP AT), CDP-diacylglycerol synthases 1 and 2 (CDS1/2), cardiolipin synthase (CRLS1) and lysocardiolipin acyltransferase 1 (LCLAT1), all genes involved in the biosynthesis and re-modelling of mitochondrial cardiolipin lipids were transcriptionally down-regulated in cytoglobin expressing cells (highlighted in red). There was no change in expression of phosphatidylglycerol phosphate synthase (PGPS) and phosphatidylglycerol-phosphate phosphatase (PGPP) was not included in the microarray.

To identify biochemical pathways for further analysis we compared the transcriptome of cytoglobin expressing and control cells. Of the 22,347 targets probed, 3346 transcripts (15%) were significantly up-regulated (FDR < 0.05) and 3055 transcripts (14%) down-regulated (FDR < 0.05). Within this set, 767 genes were up-regulated and 714 genes down-regulated by more than 1.5-fold. GO enrichment analysis of genes altered by more than 1.5-fold identified the following GO terms: NADP, oxidoreductase, oxidation–reduction process (enrichment score 6.18) and collagen catabolic process, hydroxylation and collagen (enrichment score 3.83). Next, we used KEGG pathway analysis using DAVID v6.7 to further investigate possible changes in cellular processes. Using the set of 3346 significantly up-regulated transcripts in cytoglobin expressing cells, key pathways identified included “non-alcoholic fatty liver disease” (2.2-fold enrichment) and “oxidative phosphorylation” (1.9-fold enrichment). Multiple subunits of the electron transport chain complex were shown to be up-regulated: 13 in complex I, 2 in complex II, 5 in complex III and 4 in complex IV in cytoglobin expressing cells (Fig. 1A). Specific differentially expressed genes relating to the respiratory complexes are detailed in Fig. 1B. Additionally, some genes associated with the respiratory complex were found to be up-regulated and others down-regulated, demonstrating that changes in gene expression were not a result of global regulation. Other pathways altered were “glycosaminoglycan biosynthesis” (3.6-fold enrichment), “regulation of autophagy” and “cell cycle” (1.8-fold enrichment).

Using the set of 3055 significantly down-regulated transcripts, key pathways identified included: “glycerophospholipid metabolism” (2.0-fold enrichment), “focal adhesion” (1.6-fold enrichment), and “N-glycan biosynthesis” (2.2-fold enrichment). Interestingly, changes in genes involved in glycerophospholipid metabolism (Fig. 1C) included: 1-acylglycerol-3-phosphate O-acyl transferase 3, CDP-diacylglycerol synthases 1 and 2, cardiolipin synthase and lysocardiolipin acyltransferase 1: all genes involved in the biosynthesis of mitochondrial specific cardiolipin known to regulate both electron transport chain activity and apoptosis. The cardiolipin remodelling genes *HADHA* and *TAZ* were not present on the microarray. When the data set was analysed as a whole, other additional terms identified included: “p53 signalling pathway”, “apoptosis”, “glutathione synthesis” and “fatty acid metabolism”. RT-qPCR validation of the microarray data using 8-selected genes and comparison with other cytoglobin expressing cell lines is shown in Figure S3.

### Cytoglobin expression increases cell growth and motility

**Figure 2:**
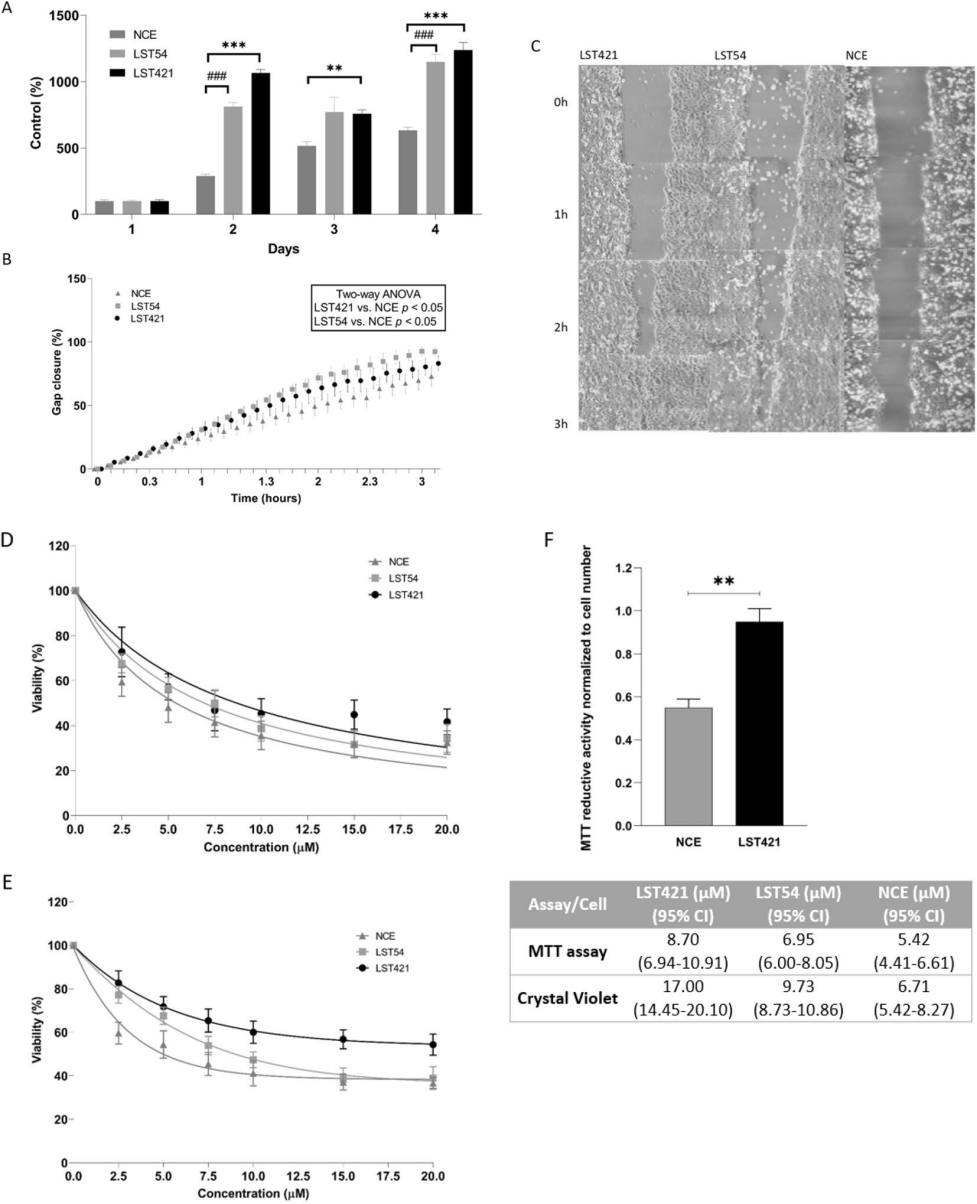
Cytoglobin expression increases cell growth and motility. (**A**) Cell number in culture following plating at the same density, (**B**,**C**) increased in cell motility as assessed by the wound healing assay and quantification using an In cell analyser. The results represent the mean of three experiments carried out in duplicate ± SD (n = 3). *** (*p* < 0.001) and ** (*p* < 0.01) represent t-test statistical significant difference between LST421 and NCE cells. ### (*p* < 0.001) represents t-test statistical significant differences between LST54 and NCE cells. Two-way ANOVA shown in **B** appear inset. Cytoglobin expression protects cells from cisplatin-induced cytotoxicity. Cells were treated with cisplatin 0–20 µM cisplatin for 24 h. (**D**) Cell viability as assessed by the MTT assay (line represents non-linear least squares regression analysis of normalised response data), (**E**) Cell viability as assessed by the crystal violet assay (line represents non-linear least squares regression analysis of normalised response data) and **F**) MTT assay normalised to cell number. Inset table contains IC_50_ values as determined using both the MTT and Crystal Violet assay calculated using least squares non-linear regression analysis. The results represent the mean of three experiments carried out in duplicate ± SD (n = 3). ** Significantly differently from un-transfected parent cell line, *p* < 0.01.

Cells expressing cytoglobin proliferated more quickly in culture (Fig. 2A), indicating that the expression of cytoglobin may initiate changes in cellular phenotype which support more rapid proliferation of the cell. Additionally, cytoglobin expressing cells also demonstrated increased motility in wound healing assays as quantified using the inCell analyser (Fig. 2B, and 2C), This effect was statistically significant (*p* < 0.01) in cells expressing both high and intermediate levels of cytoglobin.

### Cytoglobin protects cells from cisplatin-oxidative stress and toxicity

To study if cytoglobin expression protects cells from cisplatin, we assessed a panel of known biochemical endpoints related to cisplatin toxicity. Cell viability as assessed by the MTT assay (mitochondrial reductive capacity) showed a statistically significantly increase (*p* < 0.05) in cells expressing cytoglobin compared to control cells following treatment with cisplatin (0–20 µM, 48 h, Fig. 2D). Non-linear least squares regression analysis determined IC_50_ values of 8.70 µM (6.94–10.91 µM 95% CI), 6.95 µM (6.00–8.05 µM 95% CI) and 5.42 µM (4.41–6.61 µM 95% CI) in the LST421, LST54 and control cells respectively using the MTT assay. IC_50_ values were also determined using the crystal violet assay (cell number) (Fig. 2E). Non-linear least squares regression analysis demonstrated IC_50_ values of 17.00 µM (14.45–20.10 µM 95% CI), 9.73 µM (8.73–10.86 µM 95% CI) and 6.71 µM (5.42–8.27 µM 95% CI) in LST421, LST54 and control cells respectively. Both cytotoxicity assays demonstrate that expression of cytoglobin protects cells from cisplatin toxicity. Analysis by qPCR showed that cisplatin treatment had no effect on cytoglobin expression in any of the cell lines investigated (data not shown). Interestingly, normalised mitochondrial reductase activity is increased in cytoglobin expressing cells (Fig. 2F), suggesting changes to either specific mitochondrial activity or number in cells expressing cytoglobin. This is consistent with the identification of changes in expression electron transport genes identified in our microarray experiment.

**Figure 3:**
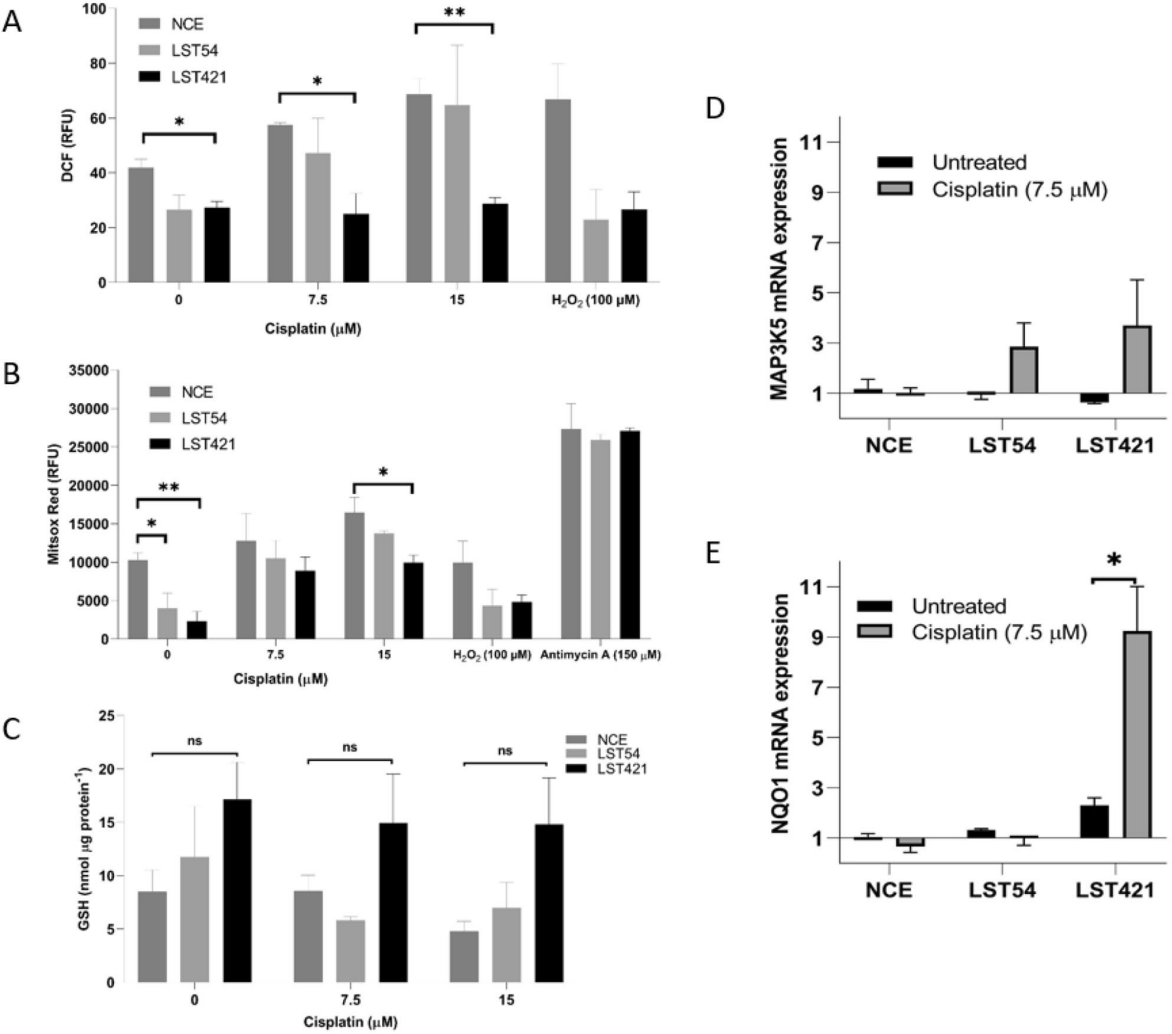
Cytoglobin expression protects cells from cisplatin mediated oxidative stress. Cells were treated with cisplatin 7.5 µ M and 15 µM cisplatin for 48 h. Hydrogen peroxide (100 µM, 1 h) and antimycin A (150 µM, 1 h) were used as positive controls. (**A**) Total levels of cellular ROS as assessed by oxidation of dichlorofluoroscein, (**B**) Mitochondrial levels of ROS as assessed by oxidation of Mitosox and (**C**) total intracellular levels of GSH. The results represent the mean of three experiments carried out in duplicate ± SD (n = 3). Unpaired t-test performed between LST421 and LST54 in comparison to the NCE cells. * represents *p* < 0.05 and ** represent *p* < 0.01. Cisplatin (7.5 µM, 48 h) regulation of (**D**) *MAP3K5* and (**E**) *NQO1* mRNA levels in control and cytoglobin expressing cells as assessed by qPCR. Wilcoxon paired test performed between the untreated and cisplatin treated cells, *p* < 0.05. The results represent the mean of three experiments carried out in duplicate ± SD (n = 3).

Quantification of reactive oxygen species (ROS) in cells showed that cytoglobin reduces levels of both total cellular ROS (Fig. 3A) and mitochondrial superoxide (Fig. 3B). Cytoglobin also protects cells from cisplatin (7.5 and 15 µM, 48 h) and hydrogen peroxide (100 µM, 1 h) induced oxidative stress (Fig. 3A,B). Consistent with the role of cytoglobin as a regulator of the intracellular redox environment, levels of reduced glutathione in cytoglobin-expressing cells were also significantly elevated (Fig. 3C). Another major cellular consequence of treatment of cancer cells with cisplatin is single and double stranded DNA breaks. As assessed by the comet assay levels of DNA-strand breaks were also significantly lower in cells expressing cytoglobin (*p* < 0.05) (Figure S4). Analysis by qPCR further confirmed changes in the cellular antioxidant response in cells expressing cytoglobin. MAP3K5 also known as apoptosis signal regulating kinase 1 (ASK1) a redox regulated stress kinase was up-regulated in cytoglobin expressing cells following treatment with cisplatin (7.5 µM, 48 h, Fig. 3D). In addition, NQO1 an anti-oxidant transcript regulated by the antioxidant response element activating transcription factor Nrf2 was significantly over-expressed in cells expressing high levels of cytoglobin (Fig. 3E). These data suggest that cells expressing cytoglobin have a greater anti-oxidant signalling response following cisplatin and this may explained the reduction in parameters related to oxidative stress observed.

**Figure 4:**
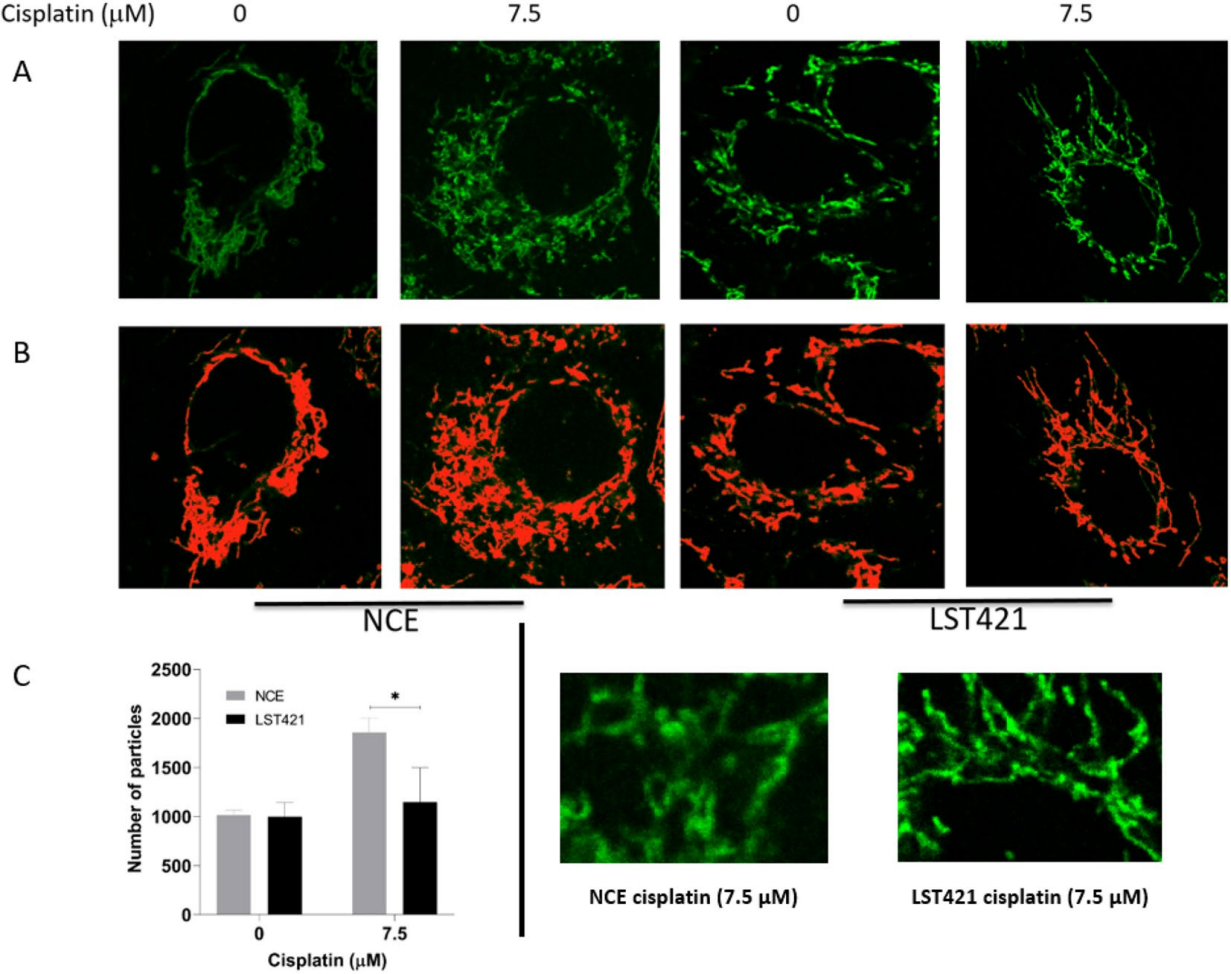
Cytoglobin protects cells from cisplatin (7.5 µM, 48 h) mediated fragmentation of mitochondria. Mitochondria were visualised by staining with mitotracker (**A**), magnified images of cisplatin treated NCE and LST421 cells are displayed in the bottom right of the figure. Threshold images were created in ImageJ (**B**) and used to quantify the number of mitochondria per cell (**C**). The results represent the mean of three independent fields of view ± SD (n = 3) with each field of view containing on average 5–10 cells. * Significantly differently from the un-transfected parent cell line, *p* < 0.05.

To further investigate changes to mitochondria, cells were stained with fluorescent mitotracker dye. In both control and cytoglobin expressing cells mitochondria demonstrated a typical elongated tubular structure. Following treatment with cisplatin (7.5 µM, 48 h), control cells contained small rounded mitochondria indicative of oxidative stress-induced mitochondrial fission (Fig. 4A,B). In contrast, in cells expressing cytoglobin there was at least partial preservation of normal mitochondrial morphology (Fig. 4A,B). This was confirmed by quantifying the number of mitochondria as discrete particles in threshold images (Fig. 4C), analysed using ImageJ.

### Cytoglobin increases levels of the apoptosis regulating mitochondrial lipid cardiolipin

**Figure 5:**
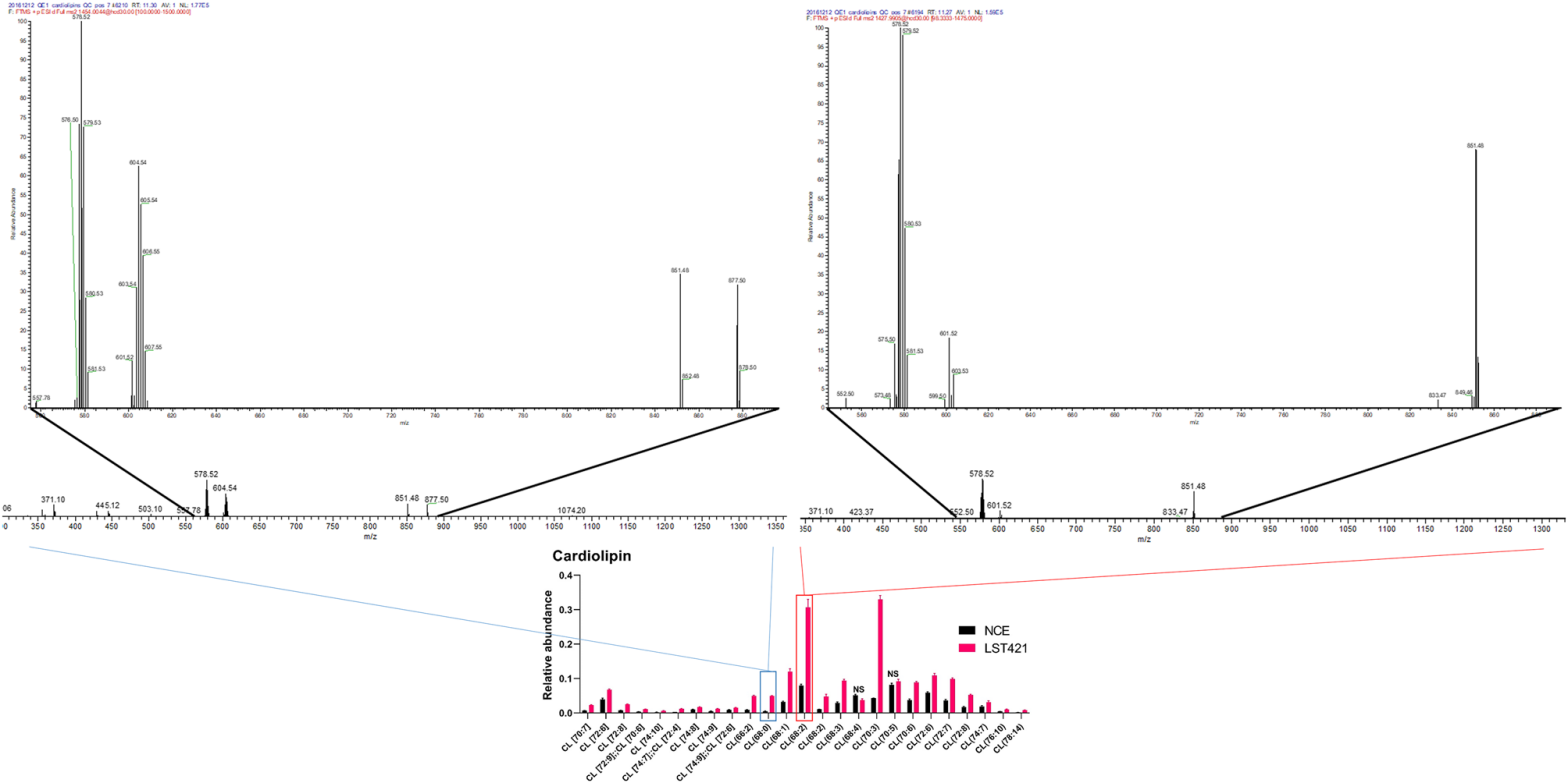
Top right; HCD MS/MS mass spectrum for a cardiolipin (CL[68:2]) detected at a mass-to-charge ratio (m/z) of 1427.9905 for the product ion demonstrating the characteristic precursor and product ions. The x-axis represents the m/z and the y-axis represents response. The figure visualises the full MS/MS spectrum (large plot) with the m/z region where product ions are detected being increased in size in the smaller insert. Top left: HCD MS/MS mass spectrum for a cardiolipin (CL[68:0]) detected at a mass-to-charge ratio (m/z) of 1454.0044 for the product ion demonstrating the characteristic precursor and product ions. The x-axis represents the m/z and the y-axis represents response. Bottom; Relative changes in the abundance of cardiolipin metabolites in NCE and cytoglobin expressing cells. All grouped columns represent significant changes between both conditions except where NS (not significant) is stated.

Next, we used mass spectrometry lipidomics to identify changes in lipid metabolism linked with cytoglobin expression, and response to cisplatin treatment. Where possible compounds were annotated by MS/MS and matched using the Thermo Fisher Lipid Search Software in silico MS/MS library (Table S1). In total, there were 1269 putative identified lipid metabolites of Glycerophospholipid, Lysoglycerophospholipid, Ceramide, Sphingolipid, Cardiolipin and included several acyl carnitines, Sterol esters and Ubiquinone metabolites. Of these, there were; 351 up-regulated and 303 down-regulated glycerophospholipids (Table S2), 11 up-regulated and 29 down-regulated lysoglycerophospholipids (Table S2), 29 up-regulated and 18 down-regulated Ceramides (Table S2), 41 up-regulated and 8 down-regulated Sphingolipids (Table S2) and 24 up-regulated and 1 down-regulated Cardiolipins (Table 1). Lipid metabolites classes identified using the MS/MS library were as follows; Phosphatidic acid (2 up-regulated and 1 down-regulated, Figure S6), Phosphatidylcholine (27 up-regulated and 16 down-regulated, Figure S6), Phosphatidylglycerol (4 up-regulated and 5 down-regulated, Figure S7), Phosphatidylinositol (2 up-regulated and 3 down-regulated, Figure S7), Phosphatidylserine (3 up-regulated and 8 down-regulated, Figure S7), Ceramide (6 up-regulated and 6 down-regulated, Figure S8) Lysophosphatidylglycerol (0 up-regulated and 10 down-regulated, Figure S9), Phosphatidylethanolamine (19 up-regulated and 31 down-regulated, Figure S10), and Sphingolipid (14 up-regulated and 0 down-regulated, Figure S11). A complete list of significantly altered metabolites is shown in Supplementary Table S2. Of the 25 significantly altered cardiolipin (Table 1 and Fig. 5), 24 were up-regulated and 1 down-regulated in CYGB expressing cells in comparison to non-CYGB (NCE) expressing cells. Additional fragmentation MS/MS mass spectra for CL [68:0] and [68:2] is displayed in Fig. 5 along with an extracted ion chromatogram for CL [74:9] in Figure S5. Extracted ion chromatogram (Figure S5) confirms upregulation of CL [74:9] in the LST421 cytoglobin expressing cells.

**Table 1:** Cardiolipin metabolites differentially regulated in cytoglobin (LST421) expressing cells compared to un-transfected control (NCE).

Metabolites	Idx	Ion	m/z	Retention time (s)	Cytoglobin expressing LST421 / NCE	q-value (FDR corrected *p* value)	Statistically significant
CL [70:7]	8427	Positive	1440.9887	643	3.32	1.2E−10	Yes
CL [72:6]	8543	Positive	1471.0352	659	1.72	0.00000299	Yes
CL [72:8]	8520	Positive	1467.0041	645	3.38	8.15E−12	Yes
CL [72:9];;CL [70:6]	8443	Positive	1447.9578	650	3.16	1.95E−09	Yes
CL [74:10]	8613	Positive	1491.0043	639	3.09	3.44E−09	Yes
CL [74:7];;CL [72:4]	8577	Positive	1480.0197	676	4.30	2.84E−10	Yes
CL [74:8]	8641	Positive	1495.0342	652	1.71	0.0000883	Yes
CL [74:9]	8632	Positive	1494.0235	647	2.68	0.000000129	Yes
CL [74:9];;CL [72:6]	8559	Positive	1475.9891	659	1.78	0.00010081	Yes
CL(66:2)	6543	Negative	1375.9623	668	5.48	3.93E−14	Yes
CL(68:0)	8468	Positive	1454.0055	678	9.65	2.94E−14	Yes
CL(68:1)	6607	Negative	1406.0039	679	3.73	2.09E−12	Yes
CL(68:2)	6604	Negative	1403.9932	679	3.89	1.93E−12	Yes
CL(68:2)	8387	Positive	1427.9906	681	4.52	9.46E−10	Yes
CL(68:3)	6597	Negative	1401.9761	666	3.26	8.61E−12	Yes
CL(68:4)	6591	Negative	1399.9607	657	0.72	0.0081287	No
CL(70:3)	6647	Negative	1430.0115	677	7.68	9.49E−17	Yes
CL(70:5)	6639	Negative	1425.9798	657	1.12	0.00073212	No
CL(70:6)	6636	Negative	1423.9647	649	2.41	0.00000046	Yes
CL(72:6)	6692	Negative	1451.9949	657	1.86	0.00000445	Yes
CL(72:7)	6685	Negative	1449.9785	653	2.75	2.48E−11	Yes
CL(72:8)	6681	Negative	1447.9632	645	3.07	1.1E−12	Yes
CL(74:7)	6760	Negative	1478.0076	662	1.68	0.034776	Yes
CL(76:10)	6821	Negative	1499.9935	645	2.37	0.00014077	Yes
CL(78:14)	6869	Negative	1519.9790	678	5.01	6.2E−12	Yes

Following treatment of cytoglobin expressing cells with cisplatin (7.5 µM, 24 h) there were 793 metabolites identified that were statistically significantly altered compared to untreated cytoglobin expressing cells. The magnitude of the differences in the expression between the two groups was smaller than between untreated non-cytoglobin expressing and untreated cytoglobin expressing cells and additionally, treatment with cisplatin resulted in a decrease in metabolite abundance but further changes to levels of cardiolipin were observed. A complete list of significantly altered metabolites during cisplatin treatment is shown in Table S2.

### Cytochrome *c* release and activation of caspase 9

**Figure 6:**
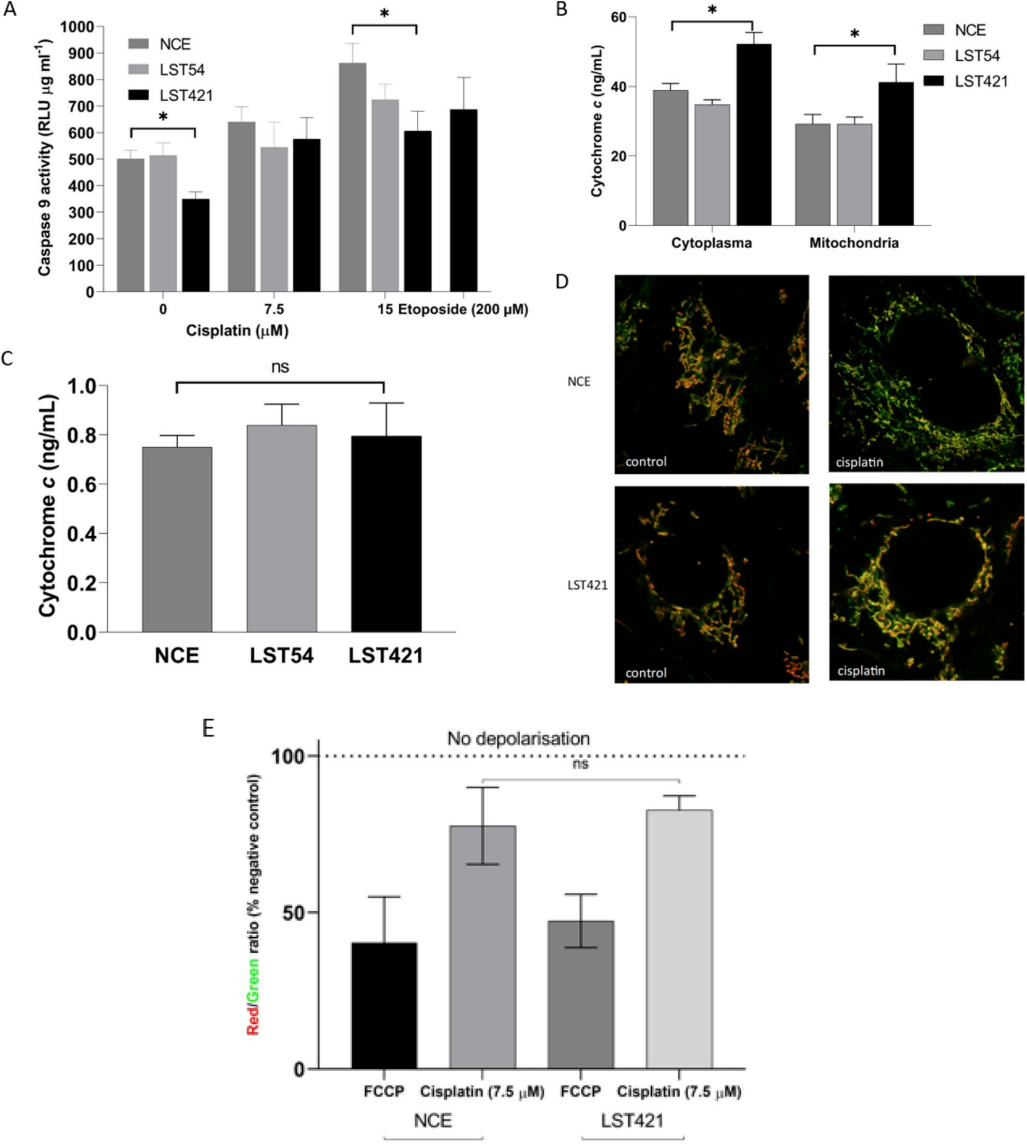
(**A**) Caspase 9 release from NCE control, LST54 and LST421 cells as measured through Caspase-Glo 9 assay kit over 0, 7.5 and 15 µM cisplatin and the positive control etoposide (200 µM). (**B**) ELISA detection of cytochrome *c* from both cytoplasmic and mitochondrial fractions in LST421, LST54 and NCE cells after cisplatin exposure (7.5 µM). (**C**) Comparison of the ratio of mitochondrial:cytoplasmic cytochrome *c* after exposure to cisplatin (7.5 µM). (**D**) Cytochrome *c* release from mitochondria using Mitotracker green and FITC 568 secondary labelled cytochrome *c*. (**E**) Mitochondrial depolarisation measured using JC-1 fluorophore in the NCE control and LST421 after exposure to FCCP (20 nM) and cisplatin (7.5 µM). Unpaired *t*-test used to determine statistical significance. * indicates a *p* < 0.05. ns = non-significant.

As shown in Fig. 6A the basal level of caspase 9 activity in cells expressing high levels of cytoglobin is significantly lower than in non-expressing control cells. In contrast, there was no significant difference between cells expressing intermediate levels of cytoglobin and controls. Following treatment of cells with cisplatin (7.5 and 15 µM) for 48 h a concentration-dependent increase in caspase 9 activity was observed in all cells. Following treatment with 15 µM cisplatin, increasing levels of cytoglobin expression resulted in a statistically significant (*p* < 0.05) decrease in caspase 9 activity (Fig. 6A). To investigate the mechanism by which cytoglobin reduces caspase 9 activity levels in cells we next studied whether cytoglobin could alter the release of cytochrome *c* from mitochondria. ELISA analysis of cytochrome *c* in cytoplasmic and mitochondrial protein determined that there were no significant changes in cytochrome *c* release between the cytoglobin expressing NCE cells, and cisplatin treatment (Fig. 6B). However, cytoglobin-expressing cells demonstrated higher total levels of cytochrome *c* expression than NCE cells (Fig. 6B). Additionally, the ratio of mitochondrial:cytoplasmic cytochrome *c* showed no significant change between LST421, LST54 and control cells (Fig. 6C) and there was only limited evidence of cytoplasmic release of cytochrome *c* as assessed by confocal microscopy (Fig. 6D). However, while the release of cytochrome *c* is relatively low in cytoglobin expressing cells, Fig. 6E shows clear depolarisation of the mitochondrial membrane when exposed to cisplatin. Additionally, the cardiolipin specific fluorescent dye 10-nonyl acridine orange demonstrates increased levels of total cardiolipin during exposure to cisplatin and increased levels in cytoglobin expressing cells in contrast to non-expressing cells (Figure S12 A and B).

### Cytoglobin and cell cycle response to cisplatin

**Figure 7:**
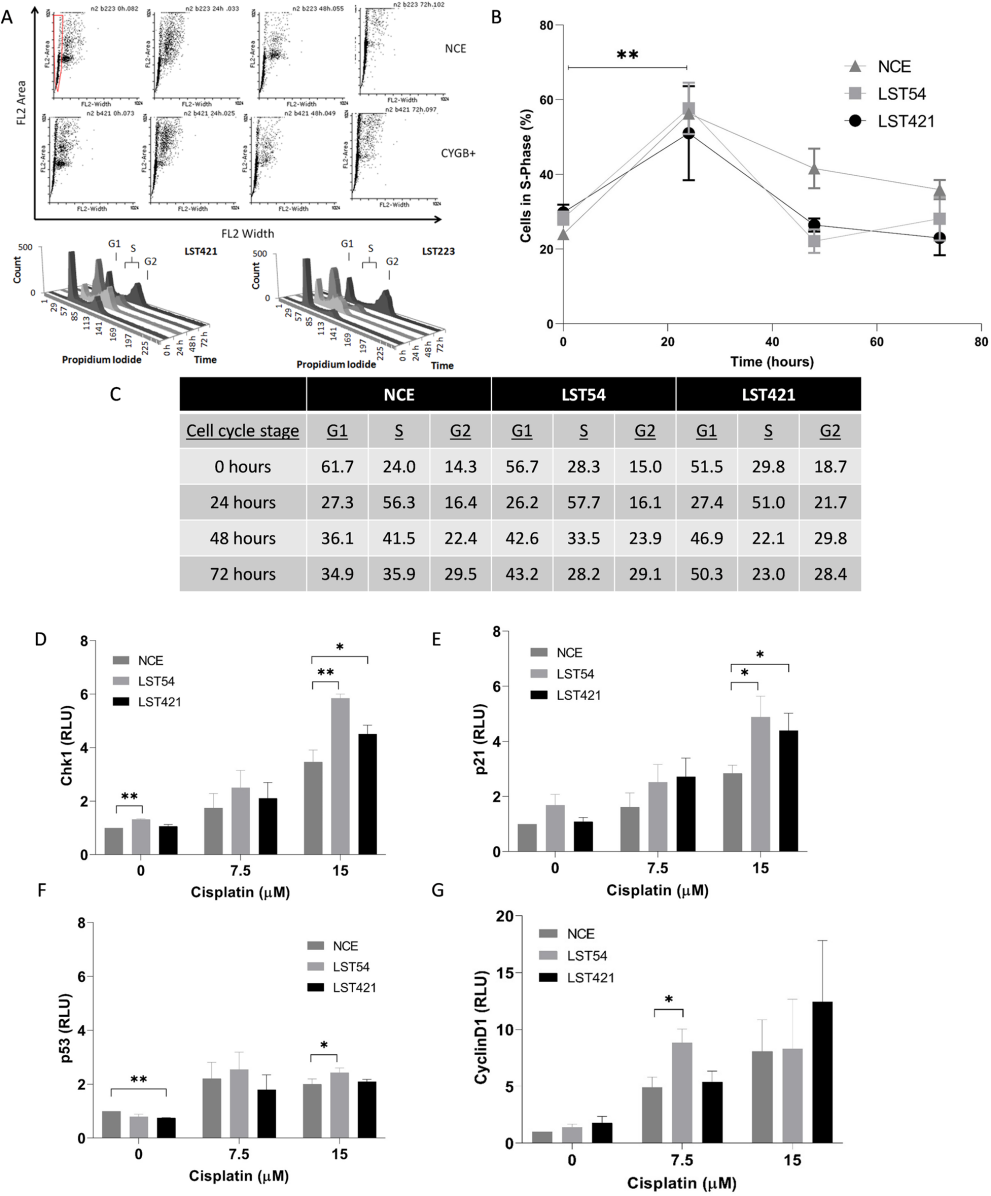
Effect of cisplatin (7.5 µM) treatment on cell cycle as assessed by propidium iodide staining and flow cytometry at 0 h, 24 h, 48 h and 72 h (**A)** distribution of propidium iodide staining among cell population, (**B)** changes in percentage of cells in S-Phase, **C**: percentage of cells in different stages of the cell cycle). Changes in protein expression of DNA damage markers; Chk1 (**D**), p21 (**E**), p53 (**F**), and cyclin D1 (**G**) after cytoglobin expressing and non-cytoglobin expressing cells were treated with cisplatin (7.5 and 15 µM). The results from (**A**–**C)** represent the mean of three experiments carried out in duplicate ± SD (n = 3). The results from (**D**–**G)** represent the mean of three independent experiments (± SD) (n = 3). Data is in normalised relative luminescence units in relation to the untreated NCE control (**D**–**G**). Statistical significance was assessed using unpaired *t*-tests with Welsh’s correction. * *p* < 0.05 and ** *p* < 0.01 in comparison to the NCE control cells.

Changes to the cell cycle are a key cellular response to stress. The time-dependent effect of cisplatin (7.5 µM) on cell cycle was therefore quantified. The percentage of untreated cells in the G1 phase of the cell cycle was significantly lower in cells expressing cytoglobin compared to non-expressing cells being: 51.5 ± 4.1%, 56.7 ± 2.8% and 61.7 ± 2.8% for LST421 (high), LST 54 (intermediate) and non cytoglobin-expressing cells respectively (Fig. 7A). Following 24 h treatment with cisplatin there was a statistically significant increase in the number of cells in S-phase in all cell types compared to untreated controls: 51.0 ± 12.0%, 57.7 ± 6.9% and 56.3 ± 1.4% for LST421 (high), LST 54 (intermediate) and non cytoglobin-expressing cells respectively (Fig. 7B,C (inset table)). However, there was no statistically significant difference in response between NCE cells and those expressing cytoglobin. At later time points, NCE cells continued to show arrest in S-phase with 41.6 ± 5.2% and 35.9 ± 4.8% of cells being in S-phase at 48 and 72 h respectively (Fig. 7B,C (inset table)). In contrast, both intermediate and high cytoglobin expressing cells (LST54 and LST421) overcame S-phase arrest with a non-statistically significant difference with the untreated controls (26.4 ± 1.8, 23.3 ± 3.1) at 48 h and (23.0 ± 4.6 and 28.2 ± 5.9) at 72 h for LST421 (high) and LST54 (intermediate) levels of expression respectively (Fig. 7C**,** inset table). The regulation of the cell cycle is strongly associated with markers of DNA damage. Cells expressing cytoglobin showed statistically significant increase in induction of the DNA-damage response proteins; Chk1 (Fig. 7D), p21 DNA (Fig. 7E), p53 (Fig. 7F) and non-significant increases in cyclin D1 (Fig. 7G), another protein involved in the G1/S-transition of cell cycle. In contrast, although cisplatin treatment resulted in minor induction of p53 (Fig. 7F) there was only small changes between the cytoglobin expressing and non-cytoglobin expressing control cell line. Cytoglobin expressing cells leave the S-phase of the cell cycle more quickly than the NCE control cell line and demonstrate enhanced DNA damage protein action. Overall, this demonstrates that cytoglobin expressing cells may be able to overcome p53-mediated checkpoint controls on the cell cycle and thereby resist potential apoptosis outcomes.

## Discussion

Cytoglobin is epigenetically silenced in some cancers^1–5,36,37^ and cytoglobin knockout mice have shown an enhanced proliferative/inflammatory phenotype^38,39^. In contrast, up-regulation of cytoglobin in other cancers including non-small cell lung carcinoma and cancers derived from the nasal and oral epithelium is also observed^4,40,41^ indicating a bimodal role of cytoglobin in cancer progression. Cytoglobin expression in a large panel of cancers has also been assessed and RNAseq data is publicly available for example in the cBIOPortal public repository (https://www.cbioportal.org/). Studies demonstrate a cyto-protective function for cytoglobin with a mechanism involving regulation of cellular redox homeostasis^12,13,16,18,42–45^. Furthermore, evidence links cytoglobin to oncogenic phenotypes^23,46^, resistance to chemotherapeutic drugs including doxorubicin and etoposide^25^, and increased tumour invasiveness^1,2,41^. In this study, transcriptomic profiling identifies cytoglobin-dependent up-regulation of genes involved with oxidative phosphorylation, regulation of autophagy and cell cycle regulation. Cytoglobin also has a role in redox homeostasis^9,36,47–51^, which if deregulated results in oxidative stress. Many of the observed transcriptional changes observed in cytoglobin-expressing cells are associated with mitochondrial function. This organelle is a major source of cellular ROS suggesting a link between cytoglobin and mitochondrial redox homeostasis. Also observed were changes in pathways related to synthesis of mitochondrial specific cardiolipins further supporting a role for cytoglobin in regulating mitochondrial function.

Electron transport chain oxidative stress-induced apoptosis is a major therapeutic mode of action of cisplatin^52^, in this study cytoglobin expressing cells have reduced levels of total cellular ROS and mitochondrial superoxide species after treatment with cisplatin. Consistent with this, cytoglobin afforded resistance to cisplatin toxicity and it is likely that either direct anti-oxidant effects or regulation of electron transport chain generation of mitochondrial ROS is the mechanistic basis of resistance to cisplatin. In addition to biochemical characterisation of oxidative stress, gene-expression analysis showed increases in expression of the oxidative stress kinase MAP3K5 and NQO1 which is transcriptionally regulated by the anti-oxidant response element (ARE). Expression of NQO1 has been previously linked to protection from cisplatin-induced nephrotoxicity through reducing oxidative stress^6^.

Previous studies have shown that cytoglobin anti-oxidant function is dependent on both aryl hydrocarbon receptor and ARE transcriptional regulation^16,17,37,47,53^, as well as direct ROS-scavenging mechanisms^13,54^. This anti-oxidant response is also linked to reduced levels of DNA-strand breaks as reported here and in previous studies^26,55^. Furthermore, DNA-damaging agents including adriamycin, etoposide and UV-light have been shown to post-transcriptional stabilise cytoglobin which has been linked to p53-dependent transcriptional activation of downstream targets including p21^26^. Up-regulation of CDKN2A, MAP3K5, and NQO1 in our cell model suggests that oxidative stress-induced p53 transcriptional activity is enhanced in cytoglobin expressing cells. Analysis of cell cycle regulation in cytoglobin expressing cells demonstrates that non-expressing cells remained in S-phase arrest after cisplatin treatment. Proteins involved in the DNA-damage response were also elevated in cytoglobin expressing cells. This included enhanced regulation of p53-controlled stress transcripts and greater induction of Chk1 and p21 proteins, both components of the ATR kinase DNA damage response pathway^56^. It has been shown that cisplatin apoptosis resistance and the G1 checkpoint could be enabled in p53 and ATR kinase-negative colorectal cells by re-introducing wildtype p53^57^. This is consistent with our findings where cytoglobin expression in p53-wildtype oral cancer cells treated with cisplatin leads to increased G1 arrest, enhanced Chk1 and p21 induction, and resistance to apoptosis. While autophagy-dependent cell death has also been recorded in relation to cisplatin induced cell death, apoptosis has been consistently reported as the major mode of cell death induced by cisplatin in head and neck cancer cells^58,59^.

Mitochondria play a pivotal role in energy metabolism, ROS production and the regulation of apoptosis. Transcriptomic profiling of cytoglobin expressing cells revealed changes in oxidative phosphorylation genes, cardiolipin synthase and lysocardiolipin acyltransferase 1, all of which affect mitochondrial energy metabolism and regulation of apoptosis. The lipidomic study in cytoglobin expressing cells showed increased level of phosphatidic acid and phosphatidylglycerol, which have been associated with cardiolipin incorporation^60^. Additionally, cardiolipin formation has been negatively associated with phosphatidylethanolamine regulation^61^. Furthermore, regulation of cardiolipin synthase, as seen in this study through transcriptional downregulation, has been shown to trigger changes in mitochondrial morphology^62^, which is consistent with the lipidomic analysis in this study. Cardiolipin are a mitochondrial specific class of lipids with a critical role in the regulation of both electron transport chain activity and inhibiting release of cytochrome *c*^63–65^. Interestingly, analysis of mitochondrial morphology showed that after treatment with cisplatin, cytoglobin protected mitochondria from changes consistent with oxidative stress-induced mitochondrial fission^66^. This shows that changes in mitochondrial function regulated by cytoglobin directly influences resistance to cisplatin. Cardiolipin and their oxidised species are a challenging class of lipid to analyse, primarily due to their mitochondrial specific location, sample processing and computational analysis of differential chain and bond positions^67,68^. In addition to the lipidomics approach taken in this study, we also utilised the cardiolipin specific dye 10-nonyl acridine orange to further examine changes in this class of lipid. Treatment with cisplatin significantly elevated the level of cardiolipin. Interestingly, cardiolipin levels were also elevated in cytoglobin expressing cells in comparison to non-expressing cells, demonstrating that increased resistance to apoptosis may be associated with mitochondrial remodelling. The remodelling of mitochondrial structure, which cardiolipin and cytochrome *c* are known to play a functional role^69,70^, is known to contribute to the stabilisation of cellular bioenergetics in response to toxic and chemical insults. It is currently unknown if cytoglobin plays a direct role in the redox balance of this event or if it interacts with cardiolipin. Alternatively, its impact on apoptosis may occur at another event upstream or downstream of this. Additionally, cristae remodelling effecting the oligomerization of the F_1_F_0_-ATP synthase has been shown to be associated with contrasting levels of phosphatidylethanolamine and cardiolipin lipid levels^61,71^, which are observed in this study. Decreases in phosphatidylethanolamine lipid metabolites observed in cytoglobin expressing cells have also been associated with greater propensity for mitochondrial stability^61,72^*,*We believe this finding provides the mechanistic basis for the observed changes in mitochondrial structure and function.

Previous studies have also found evidence of cytoglobin association with the inhibition of apoptosis^10,49,73–75^. Cytoglobin is reported to interact with, and regulate the redox status of cardiolipin lipids^76^. Therefore, cytoglobin could directly control the release of cytochrome *c* from mitochondria regulating the sensitivity of cells to oxidative-stress induced apoptosis. Alternatively, cytoglobin may reduce apoptosis through redox-control of mitochondrial permeability transition pore opening, since superoxide-induced cytochrome *c* release has been found to be impaired by inhibitors of voltage dependent anion channel (VDAC)^77^. Cytochrome *c* is also directly redox-regulated and oxidation by hydrogen peroxide promotes apoptosis^78^. It is possible therefore that the anti-oxidant properties of cytoglobin could raise the threshold of mitochondrial ROS required to cause cytochrome C oxidation and release.

In conclusion, we present evidence that cytoglobin expression results in important phenotypic changes to oral carcinoma cells and confers resistance to cisplatin-induced apoptosis. Transcriptomic, lipidomic and mechanistic studies show that the mechanism of resistance is related to reduced levels of cellular and mitochondrial ROS, expression of electron transport genes and changes in mitochondrial cardiolipins. Our results increase the understanding of cytoglobin’s cellular function which to date has remained elusive. Furthermore, they also show that targeting cytoglobin and cardiolipins could enhance the therapeutic effectiveness of cisplatin in patients with head and neck cancer, a disease for which chemo-resistance remains a major therapeutic challenge.

## Materials and methods

### Cell culture and transfection

PE/CA-PJ41 were obtained from The European Collection of Authenticated Cell Cultures (ECACC, catalogue number 98020207) and maintained at 37 °C, 5% CO_2_ in RPMI 1640 media supplemented with 10% (v/v) foetal bovine serum, 2 mM L-glutamine, 100 U/mL penicillin and 100 µg/mL streptomycin. All cell cultures were confirmed free from *Mycoplasma sp*. contamination by PCR and cultures were maintained up to passage number 20. For cytoglobin over-expressing cell line generation, PE/CA-PJ41 cell lines were transfected with the pCMV6-Neo plasmid containing the human cytoglobin cDNA sequence as described in Figure S1.

### Haem quantification

Total levels of cellular haem containing proteins were quantified using the pyridine haemochromogen assay by measuring absorbance at 557 nm and 575 nm wavelengths^79^. Haem concentration was normalised to total protein as determined by the Bradford assay^80^.

### Quantification of oxygen consumption and intracellular ATP

The MicroRespiration System (Unisense, Denmark) was used to quantify oxygen consumption in cells (5 × 10^4^ in 1 mL of media) and oxygen consumption expressed as μmolL^−1^ h^−1^. The mitochondrial ToxGlo assay (Promega) was used to quantify levels of total adenosine triphosphate (ATP) using 3.8 × 10^4^ cells per well and levels of ATP were expressed as µmol per mg of total protein.

### Whole genome cDNA microarray and bioinformatics analysis

RNA (50 ng) from biological triplicates of control and cytoglobin expressing cells (LST421) were labelled with cyanine 5 and 3, respectively, and cRNA (300 ng) hybridised to an Agilent SurePrint G3 Human Gene Expression 8 × 60 K v1 Microarray. Quantile normalisation was performed on the 1-colour data, followed by principle component analysis in order to identify the variance of the cytoglobin vs. the non-cytoglobin expressing cells. *t*-testing was performed with Bonferroni-Hochberg correction with a False Discovery Rate (FDR) set at 5%. The resulting analysis demonstrated that 6401 transcripts were significantly different between the cytoglobin expressing and NCE control. Additionally, a cut off value of a twofold change was applied to transcripts that were statistically significantly changed. In the cytoglobin expressing cells, 272 transcripts were significantly upregulated with a fold change of 2 or higher. Additionally, in cytoglobin expressing cells 227 transcripts were significantly downregulated with a fold change of 2 or higher. Gene ontological analysis was performed using Large-Scale State Transitions genomic analysis in PANTHER v.10 software. The microarray data is archived on the public repository GEO database (GEO:GSE130441). RT-qPCR validation of the microarray data using 8 selected genes and comparison with other cytoglobin expressing cell lines is shown in Figure S3.

### Cell Motility assay and proliferation assays

Cells (4 × 10^4^ cells mL^−1^) were seeded in 24-well culture insert µ-chambers (Ibidi, Germany) and allowed to adhere overnight under standard culture conditions. The next day, inserts were removed and gap closure over 6 h was quantified using Cell IQ software to calculate percentage wound closure.

### Quantification of cisplatin cytotoxicity and caspase 9 activation

Cytotoxicity was assessed as described previously using the MTT^81^ and crystal violet^82^ assays. Caspase 9 activity was quantified using Caspase-Glo 9 Assay (Promega) according to the manufacturer's instructions, and luminescence was quantified with an Infinite 200 Pro microplate reader and normalised to total protein.

### Analysis of total cellular ROS, mitochondrial superoxide and reduced glutathione levels

Total cellular ROS and mitochondrial superoxide levels were measured using the redox sensitive dyes 2′,7′-dichlorodihydrofluoroscin-diacetate (H_2_DCFDA, Invitrogen) and Mitosox Red (Life Technologies), respectively. Total levels of cellular GSH were quantified as described previously^83^ and GSH levels were expressed as nmoles per µg protein.

### 10-Nonyl Acridine Orange staining for cardiolipin

10-Nonyl Acridine Orange has previously been used to measure the level of the mitochondrial specific lipid cardiolipin^84,85^. Cells were seeded at 3 × 10^5^ cells in 6-well plates and allowed to attach for 24 h. The next day the media was changed and cells incubated for 15 min with N-nonyl acridine orange (100 ng/mL). Following incubation, the cells were washed with PBS (1 mL) and detached using a TrypLE Express. Cells were then re-suspended in 1 mL of PBS and analysed immediately by flow cytometry (Attune NxT, Thermo Scientific). Excitation was set at 488 nm and emission recorded at 530 nm. Mean fluorescence was calculated using FlowJo software (Becton Dickinson, USA).

### Analysis of mitochondrial depolarisation using JC-1

JC-1 is a potentiometric mitochondrial membrane-permeant dye used to monitor mitochondrial membrane potential. Cells were seeded at 3 × 10^5^ cells in 6-well plates and allowed to attach for 24 h. Cell were exposed to cisplatin (7.5 µM) and FCCP (20 nM, 30 min prior to harvesting). Cells were washed with PBS, detached from the plate surface with TrypLE Express and stained with JC-1 (100 nM) for 15 min. Both the monomer and J-aggregate of JC-1 were analysed immediately using flow cytometry (Attune Nxt, Thermo Scientific) at 529 nm and 590 nm respectively. Ratiometric analysis was performed using FlowJo software (Becton Dickinson, USA).

### Quantitative real-time PCR

Total RNA was isolated using an RNeasy Miniprep Kit (Qiagen) according to the manufacturer’s instructions and 500 ng was used for cDNA synthesis. For RTqPCR, cDNA template (25 ng), 1 µL SYBR-Green primers, 10 µL PrecisionPlus qPCR 2 × mastermix (Primer Design, Southampton) and Nuclease-Free Water (Qiagen) were used in a 20 µL reaction volume. A standard 2-step protocol (1 cycle of 10 min at 95 °C and 50 cycles of 15 s at 95 °C and 30 s at 60 °C). Fold changes were calculated using the efficiency-corrected ΔΔCt method described by Pfaffl^86^ and normalised to two housekeeping genes; TATA-binding protein (TBP) and β-2-microglobulin (B2M).

### Lipidomics sample preparation and UHPLC-MS analysis

LST421 and NCE cells were seeded at 3 × 10^5^ cells in 6-well plates and allowed 24 h to attach prior to treatment with cisplatin (7.5 µM) or untreated cell culture media for 24 h followed by quenching, harvesting and extraction of the intracellular metabolome using methanol water extraction. Ultra High Performance Liquid Chromatography-Mass Spectrometry data acquisition was performed using a Dionex UltiMate 3000 Rapid Separation LC system (Thermo Fischer Scientific, USA) coupled with a heated electrospray Q Exactive Focus mass spectrometer (Thermo Fisher Scientific, USA). Data were acquired in positive and negative ionisation mode separately within the mass range of 150 – 2000 m/z at resolution 70,000 (FWHM at m/z 200). Quality control (QC) samples were analysed as the first ten injections and then every sixth injection with two QC samples at the end of the analytical batch. Deconvolution of raw data was performed with XCMS software according to the following settings of Min peak width (4 for HILIC and 6 for lipids); max peak width (30); ppm (12 for HILIC and 14 for lipids); mzdiff (0.001); gapInit (0.5 for HILIC and 0.4 for lipids); gapExtend (2.4); bw (0.25); mzwid (0.01) as described in Smith et al.^87^. Putative annotation of metabolites or metabolite groups was performed by applying the PUTMEDID-LCMS workflows operating in the Taverna workflow environment^88^. Areas under the Receiver Operator Curves (AUROC) were calculated in MetaboAnalyst with multiple metabolites combined^89^. All molecules were annotated according to guidelines for reporting of chemical analysis results, specifically to Metabolomics Standards Initiative level 2^90^. Chemical standards were not included to determine the expected retention times because of the prohibitive costs. Data was normalised to sample total peak area and defined as normalized concentration, glog transformation was performed prior to data analysis in the software MetaboAnalyst^89^. All statistical analyses are reported following correction for multiple testing applying the Benjamin-Hochberg method with FDR correction set at 5%. The cut-off values was set at a –Log(corrected p-value) of 1.3 for all lipid metabolites. Full details of Lipidomics samples preparation and UHPLC-MS analysis are presented in the SI.

### Quantification of cytochrome *c* release

Mitochondria were isolated using the reagent method in the mitochondrial isolation kit (Thermo Scientific) and cytoplasmic proteins were extracted using the NE-PER nuclear and cytoplasmic extraction reagents (Thermo Scientific). Cytochrome *c* was detected in both fractions of each sample using a cytochrome *c* ELISA kit (Novus Biologicals) according to the manufacturer’s instructions.

### In-cell ELISA

Fixed cells were permeabilised with 0.01% Triton X-100 and blocked with 3% bovine serum albumen (BSA) for 1 h. Wells were probed with mouse monoclonal antibodies against human Chk1, p21, cyclin D1 (Santa-Cruz), p53 (Life Technologies) and cytoglobin (Abnova) at 1:500 diluted and then incubated with goat anti-mouse HRP-conjugated antibody (1:1000). SigmaFast OPD Substrate (Sigma) was used for visualisation with absorbance measured at 492 nm.

### Cell cycle analysis

Cells were fixed in ethanol and stained with propidium iodide and analysed by flow cytometry as described previously^91^.

### Alkaline comet assay

Single strand DNA-breaks were assessed using the alkaline comet assay as described previously^92^. Median values of three separate experiments were analysed using ANOVA and post-hoc Student's t-test, as reported previously^93^.

## Supplementary Information

Supplementary Information

Supplementary Information
