# Supplementary material for: Cytoglobin protects cancer cells from apoptosis by regulation of mitochondrial cardiolipin
Source: Sci Rep. 2021 Jan 13;11:985. doi: 10.1038/s41598-020-79830-w (PMC7806642; doi:10.1038/s41598-020-79830-w)
Supplement: Supplementary file 2 — Supplementary Information [file 41598_2020_79830_MOESM2_ESM.docx]

**Supplementary Information (SI)**

**Cytoglobin protects cancer cells from apoptosis by regulation of mitochondrial cardiolipin**

Lorna S Thorne^1†^, Garret Rochford^1†^, Timothy D Williams^1^, Andrew Southam^1,2^, Giovanny Rodriguez-Blanco ^1,2^, Warwick B Dunn^1,2,3^, Nikolas J Hodges ^1^*

^1^ School of Biosciences, University of Birmingham, Edgbaston, Birmingham, B15 2TT, UK

^2^ Phenome Centre Birmingham, University of Birmingham, Edgbaston, Birmingham, B15 2TT, UK

^3^ Institute of Metabolism and Systems Research, University of Birmingham, Edgbaston, Birmingham, B15 2TT, UK

† Both authors contributed equally to this work

*Corresponding author

Tel: +44 (0) 121 4145906

Email: [N.Hodges@bham.ac.uk](mailto:N.Hodges@bham.ac.uk)

**Lipidomics sample processing, data acquisition and analysis**

*Sample collection and extraction:* NCE control and the cytoglobin expressing LST421 cells were seeded at 3 x 10^5^ cells in 6-well plates and allowed 24 h to attach prior to treatment with 7.5 µM of cisplatin. Untreated and treated cells were incubated an additional 24 h followed by quenching, harvesting and extraction of the intracellular metabolome as described. Cell culture media was removed and cells washed twice with 2 mL 0.9 % NaCl while on ice. NaCl was removed and 600 µL of methanol:water (2:0.8) was added to the wells, a cell scrapper was used to dislodge cells and cells/extraction solution were transferred into glass vials on ice. An additional 240 µL of methanol:water (2:0.8) was added to the wells to collect remaining cells followed by transfer in to the glass vial. A Hamilton syringe was used to add 600 µL CHCl_3_ to the glass vial followed by a vortex mixing for 30 seconds. 300 µL of ice-cold H_2_O was added to the vials and was vortex mixed for 30 seconds. Vials were incubated on ice for 10 minutes followed by centrifugation (2,500 x g for 15 min at 4 ˚C). Samples were incubated at room temperature for 5 minutes. A Hamilton syringe was used to remove the entire upper polar layer which was removed. A separate Hamilton syringe was used to remove the entire lower non-polar layer and was transferred to a fresh vial. Non-polar samples were dried under a stream of nitrogen with no heat applied.

*UHPLC-MS data acquisition:* The samples were analysed applying a Ultra High Performance Liquid Chromatography-Mass Spectrometry (UHPLC-MS) method using a Dionex UltiMate 3000 Rapid Separation LC system (Thermo Fisher Scientific, MA, USA) coupled with a heated electrospray Q Exactive Focus mass spectrometer (Thermo Fisher Scientific, MA, USA). Non-polar extracts were analysed on a Hypersil GOLD column (100 x 2.1mm, 1.9 µm; Thermo Fisher Scientific, MA, USA). Mobile phase A consisted of 10 mM ammonium formate and 0.1% formic acid in 60% acetonitrile/water and mobile phase B consisted of 10 mM ammonium formate and 0.1% formic acid in 90% propan-2-ol/water. Flow rate was set for 0.40 mL.min^-1^ with the following gradient: t=0.0, 20% B; t=0.5, 20% B, t=8.5, 100% B; t=9.5, 100% B; t=11.5, 20% B; t=14.0, 20% B, all changes were linear with curve = 5. The column temperature was set to 55 °C and the injection volume was 2μL. Data were acquired in positive and negative ionisation mode separately within the mass range of 150 – 2000 m/z at resolution 70,000 (FWHM at m/z 200). Ion source parameters were set as follows: Sheath gas = 50 arbitrary units, Aux gas = 13 arbitrary units, sweep gas = 3 arbitrary units, Spray Voltage = 3.5kV, Capillary temp. = 263 °C, Aux gas heater temp. = 425 °C. Data dependent MS2 in ‘Discovery mode’ was used for the MS/MS spectra acquisition using following settings: resolution = 17,500 (FWHM at m/z 200); Isolation width = 3.0 m/z; stepped normalised collision energies (stepped NCE) = 20, 50, 80%. Spectra were acquired in three different mass ranges: 200 – 400 m/z; 400 – 700 m/z; 700 – 1500 m/z. A Thermo ExactiveTune 2.8 SP1 build 2806 was used as instrument control software in both cases and data were acquired in profile mode. Quality control (QC) samples were analysed as the first ten injections and then every sixth injection with two QC samples at the end of the analytical batch. Two blank samples were analysed, the first as the 6th injection and then the second at the end of each batch.

*Raw data processing and metabolite annotation:* Raw data acquired in each analytical batch were converted from the instrument-specific format to the mzML file format applying the open access ProteoWizard software. Deconvolution was performed with XCMS software according to the following settings of Min peak width (4 for HILIC and 6 for lipids); max peak width (30); ppm (12 for HILIC and 14 for lipids); mzdiff (0.001); gapInit (0.5 for HILIC and 0.4 for lipids); gapExtend (2.4); bw (0.25); mzwid (0.01)^88^. A data matrix of metabolite features (*m/z*-retention time pairs) vs. samples was constructed with peak areas provided where the metabolite feature was detected for each sample. Putative annotation of metabolites or metabolite groups was performed by applying the PUTMEDID-LCMS workflows operating in the Taverna workflow environment. We applied 5 ppm mass error and a retention time range of 2 s in feature grouping and molecular formula and metabolite matching. As different metabolites can be detected with the same accurate *m/z* (for example, isomers with the same molecular formula), multiple annotations could be observed for a single detected metabolite feature. Also, a single metabolite could be detected as multiple molecules, particularly as a different type of ion (e.g., protonated and sodiated ions). Throughout this article, the term “metabolite” refers to either single metabolites or groups of molecules with the same retention time and the same accurate *m/z*. All molecules were annotated according to guidelines for reporting of chemical analysis results, specifically to Metabolomics Standards Initiative level 2^91^.

*Quality control data checks and data analysis:* The data for pooled QC samples were applied to perform QC filtering. For each metabolite feature detected QC samples were removed and the relative standard deviation and percentage detection rate were calculated. Metabolite features with a RSD > 30% and a percentage detection rate < 60% were deleted from the dataset. Missing values in the data were replaced by values applying k nearest neighbour (kNN) missing value imputation followed by normalisation to sample total peak area (as a percentage), glog transformation and Pareto scaling prior to data analysis. Principal Components Analysis (PCA) was then performed to assess the technical variability (measured by the replicate analysis of a pooled QC sample) and biological variability as part of the quality control process. Areas under the Receiver Operator Curves (AUROC) were calculated in MetaboAnalyst with multiple metabolites combined^90^. Data were normalised to sample total peak area (as a percentage) and were defined as normalized concentration (%), no missing value imputation or scaling was performed and glog transformation was performed prior to data analysis in the software MetaboAnalyst^90^. All statistical analyses are reported following correction for multiple testing applying the Benjamin-Hochberg method.

**List of Supplementary Figures and Tables**

**Figure S1:** Generation of stable PE/CA-PJ41 cell lines expressing different levels of cytoglobin. **A**) PCR screening of genomic incorporation of transgene, **B**) qPCR assessment of mRNA expression levels and **C**) In-cell ELISA. The HEK293 CYGB+ cell line is a previous generated cell line that was used as a positive control and the normal oesophageal cell line (NE-1) was also included as a reference as it is known to express cytoglobin endogenously. Three cells with low (LST32), medium (LST54) and high (LST421) were selected for further analysis. ** and * represent significantly from un-transfected parent cell line, *p* < 0.01 and 0.05 respectively HEK293 CYGB+, an immortalised human embryonic kidney cell line (previously generated in our lab) was used as a positive control for the CYGB transfection process and the primary cell NE-1, an immortalised normal oesophageal cell lines which was a gift from Dr Janet Risk (The University of Liverpool, UK) was used to demonstrate physiological level expression of CYGB. HEK293 CYGB+ were cultured similarly to PE/CA-PJ41 (Materials and Methods), except in DMEM. NE-1 were cultured in keratinocyte serum-free medium supplemented with bovine pituitary extract and EGF 1-53 (Gibco). using Turbofectin 8.0 (Origene, United States). Stable clones were selected using the cloning ring method in G418 sulfate. Genomic integration of the vector was confirmed by PCR using a Phusion High-Fidelity Kit (NEB) and the following cycling conditions 1 cycle of 98 °C for 30 sec; 30 cycles of 98 °C for 10 sec, 64 °C for 30 sec and 72 °C for 1 min; 1 cycle of 72 °C for 10 min with the following primers cygbF (5´-CCACCGCCGCCGCCGAGCAAA), cygbR (5´-TGGGGGCTCCGCTCCATCTCCA), NeoF (5´-TGGCCACGACGGGCGTTCCTTG) and NeoR (5´- GCAGCCGCCGCATTGCATCAG).

**Figure S2**: Biochemical analysis of cytoglobin-expressing PE/CA-PJ41 cells. A) Total levels of heme protein (nM/ng total protein), B) intracellular ATP concentration (µg/mg of total protein) and C) Oxygen consumption (µmol/h). The results represent the mean of three experiments carried out in duplicate ±SD (n=3).

**Figure S3**: qPCR validation of microarray data using the genes *CDKN2A,* *ITGA2,* *MAP3K5,* *ARHGAP18,* *NQO1,* *COX7C,* *BNIP3L* and *GADD45A* from RNA isolated from control and cytoglobin expressing cell lines (LST421, LST54 and LST32). Data was normalised to the average of *TBP* and *β2M* expression using the Pfaffl ΔΔCt method from Ct values averaged across three biological replicates [85]. The value in blue on each figure is the mean fold change observed between control and LST421 cytoglobin expressing cells for that gene in the microarray data.

**Figure S4:** DNA strand breaks as assessed by the alkaline comet assay. **A)** Control (NCE) and cytoglobin expressing (LST421) cells following treatment with cisplatin (7.5 µM, 24 and 48 h). The results represent the mean of three experiments carried out in duplicate ±SD (n=3). *Significantly differently from un-transfected parent cell line, *p* < 0.05. **B)** Representative images of individual comet nuclei of untreated (0 h) cytoglobin expressing (LST421) and non-expressing cells (NCE).

**Figure S5:** Extracted ion chromatograms (1475.97-1476.01) representing the detected cardiolipin (CL[74:9]. The x-axis represents chromatographic retention time and the y-axis represents response. The top three chromatograms represent samples from NCE control cells and the bottom three chromatograms represent samples from cytoglobin expressing LST421 cells. The peak height for each chromatogram is included to demonstrate the response differences between NCE and LST421 cells.

**Figure S6:** Relative changes in the abundance of phosphatidic acid (PA) and Phosphatidylcholine (PC) metabolites confirmed with MS/MS spectra in NCE and cytoglobin expressing cells. All grouped columns represent statistically significant changes except for those marked with NS (not significant). Individual expression values and associated statistics are present in **Table S2**.

**Figure S7:** Relative changes in the abundance of Phosphatidylglycerol (PG) metabolites confirmed with MS/MS spectra in NCE and cytoglobin expressing cells. All grouped columns represent statistically significant changes except for those marked with NS (not significant). Individual expression values and associated statistics are present in **Table S2**.

**Figure S8:** Relative changes in the abundance of Ceramide metabolites confirmed with MS/MS spectra in NCE and cytoglobin expressing cells. All grouped columns represent statistically significant changes except for those marked with NS (not significant). Individual expression values and associated statistics are present in **Table S2**.

**Figure S9:** Relative changes in the abundance of Lysoglycerophospholipid metabolites confirmed with MS/MS spectra in NCE and cytoglobin expressing cells. All grouped columns represent statistically significant changes except for those marked with NS (not significant). Individual expression values and associated statistics are present in **Table S2**.

**Figure S10:** Relative changes in the abundance of Phosphatidylethanolamine (PE) metabolites confirmed with MS/MS spectra in NCE and cytoglobin expressing cells. All grouped columns represent statistically significant changes except for those marked with NS (not significant). Individual expression values and associated statistics are present in **Table S2**.

**Figure S11:** Relative changes in the abundance of Sphingolipid metabolites confirmed with MS/MS spectra in NCE and cytoglobin expressing cells. All grouped columns represent statistically significant changes except for those marked with NS (not significant). Individual expression values and associated statistics are present in **Table S2**.

**Figure S12: A** Cardiolipin levels measured through Acridine Orange 10-nonyl bromide fluorescence in NCE control, LST54 and LST421 cells, untreated and after cisplatin exposure (7.5 µM). Unpaired *t*-test used to determine statistical significance. * indicates a *p* < 0.05. ns = non-significant. **B** Flow cytometry scatter plot and associated histogram of fluorescence generated using 10-Nonyl Acridine Orange on NCE and cytoglobin expressing cells. Scatter plot and histogram x-axis represents fluorescence at 530 nm and the y-axis represents side scatter height. Histograms y-axis represents incidence count.

**Table S1:** Differentially regulated metabolites in cytoglobin (LST421) expressing cells compared to un-transfected control cells, which were confirmed with MS/MS spectra. A complete list of significantly altered metabolites shown in Table S2.

**Table S2**: Spreadsheet containing putative and confirmed MS/MS ion spectra identifications along with associated relative fold change of experimental conditions (NCE, cytoglobin expressing LST 421, NCE cells treated with cisplatin and cytoglobin expressing LST421 cells treated with cisplatin) and statistical significance testing.


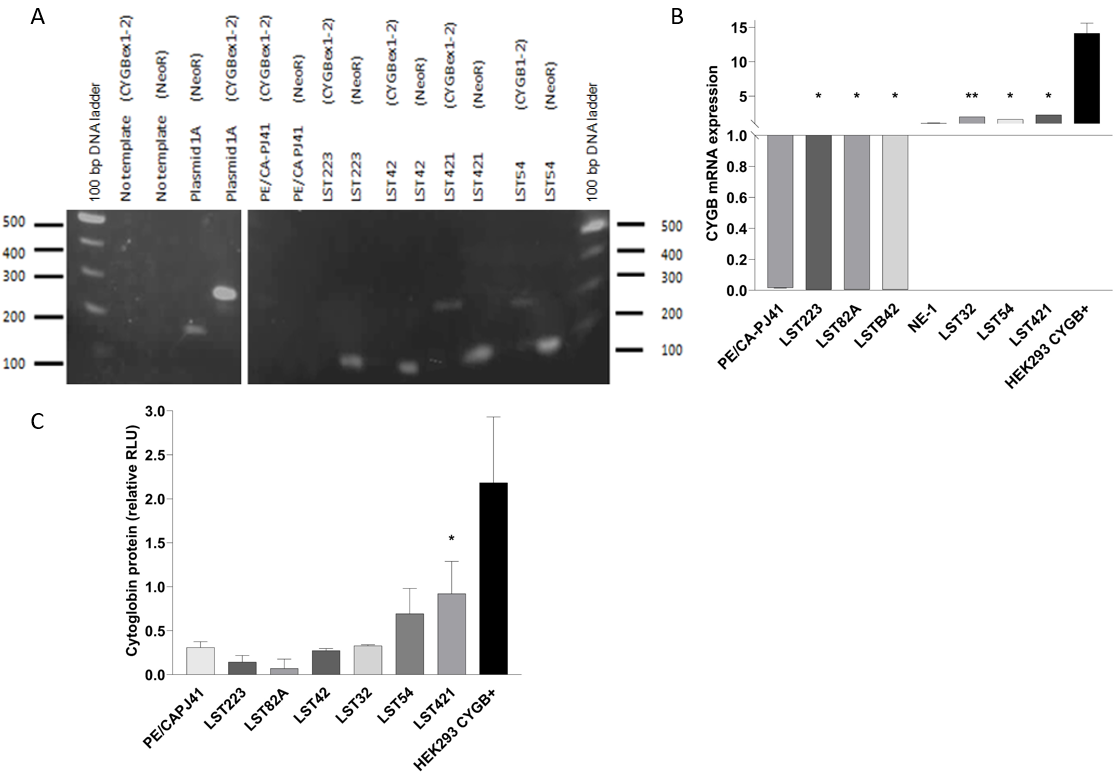


**Figure S1**


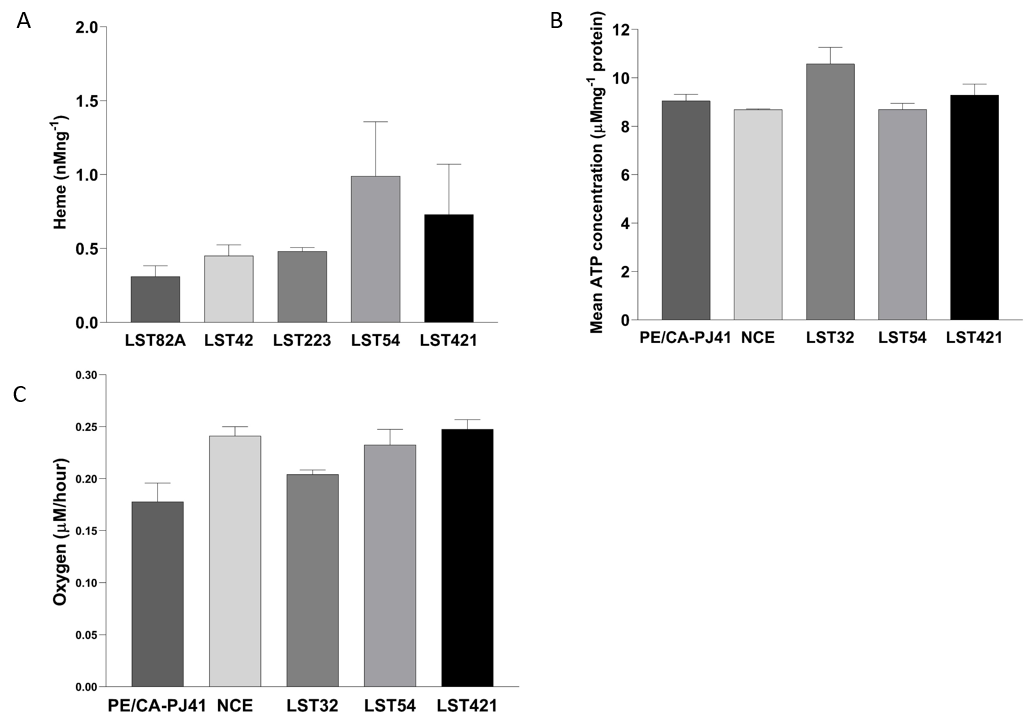


**Figure S2**


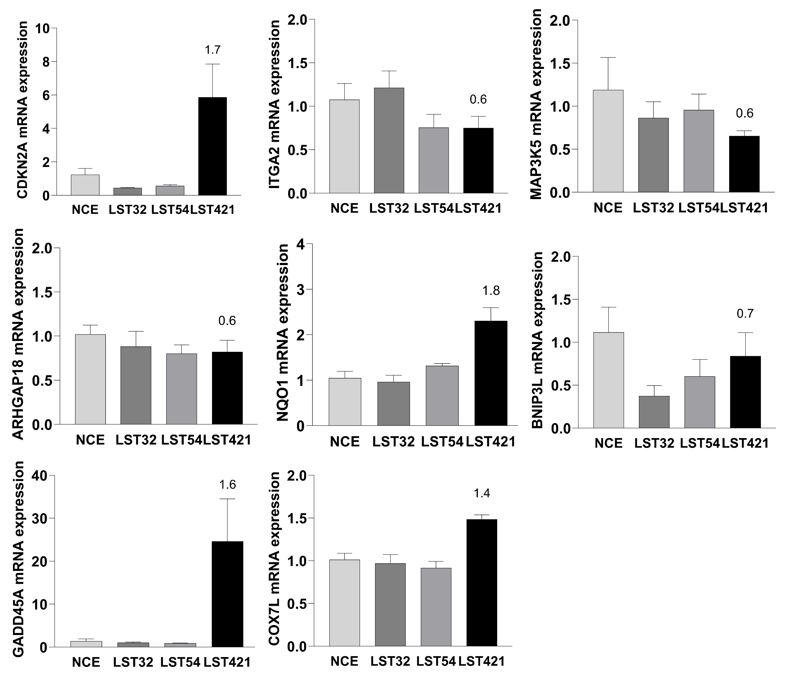


**Figure S3**


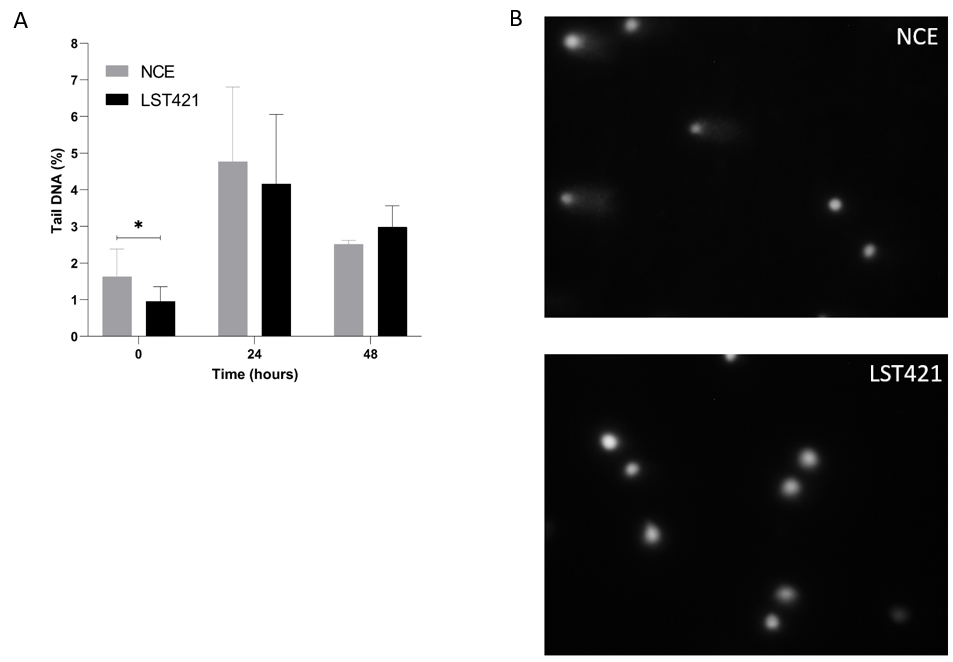
**Figure S4**


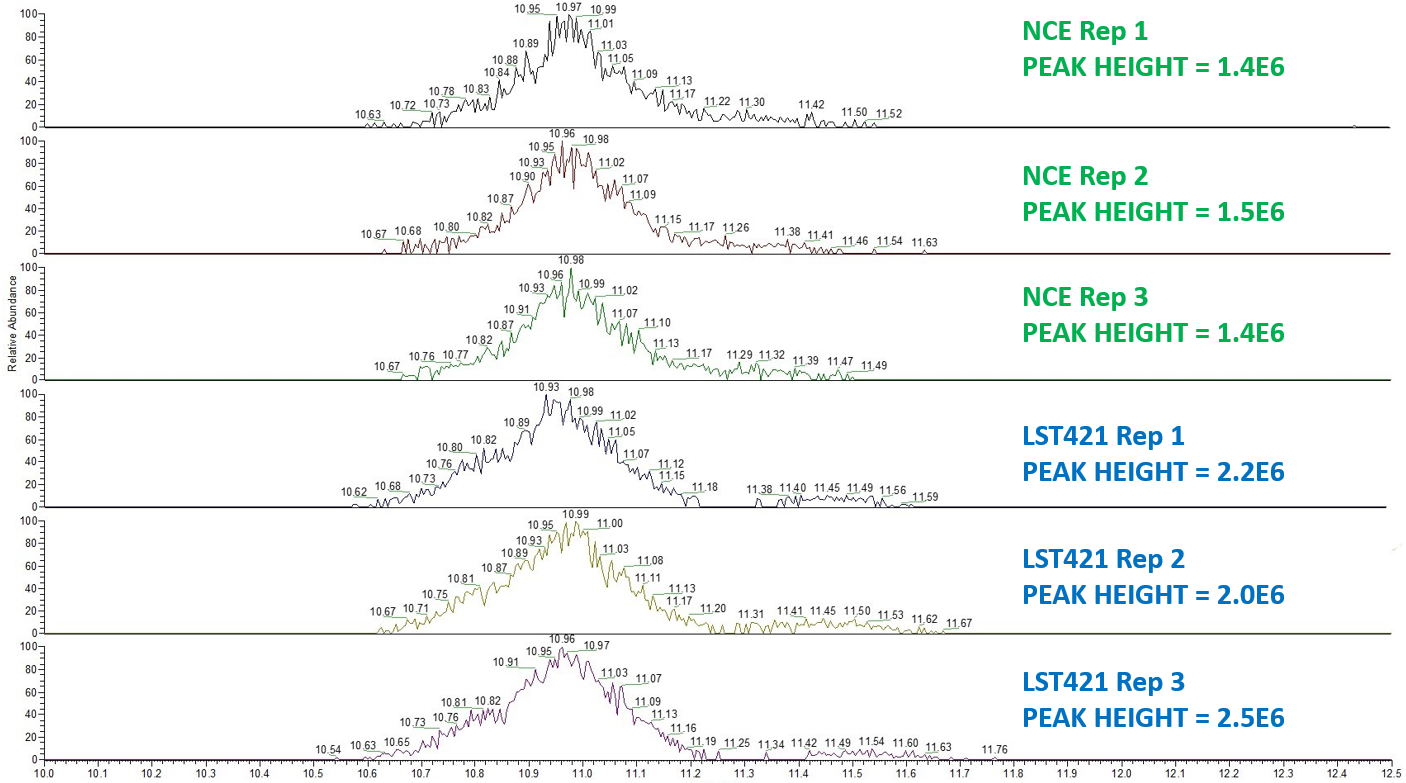


**Figure S5**

**
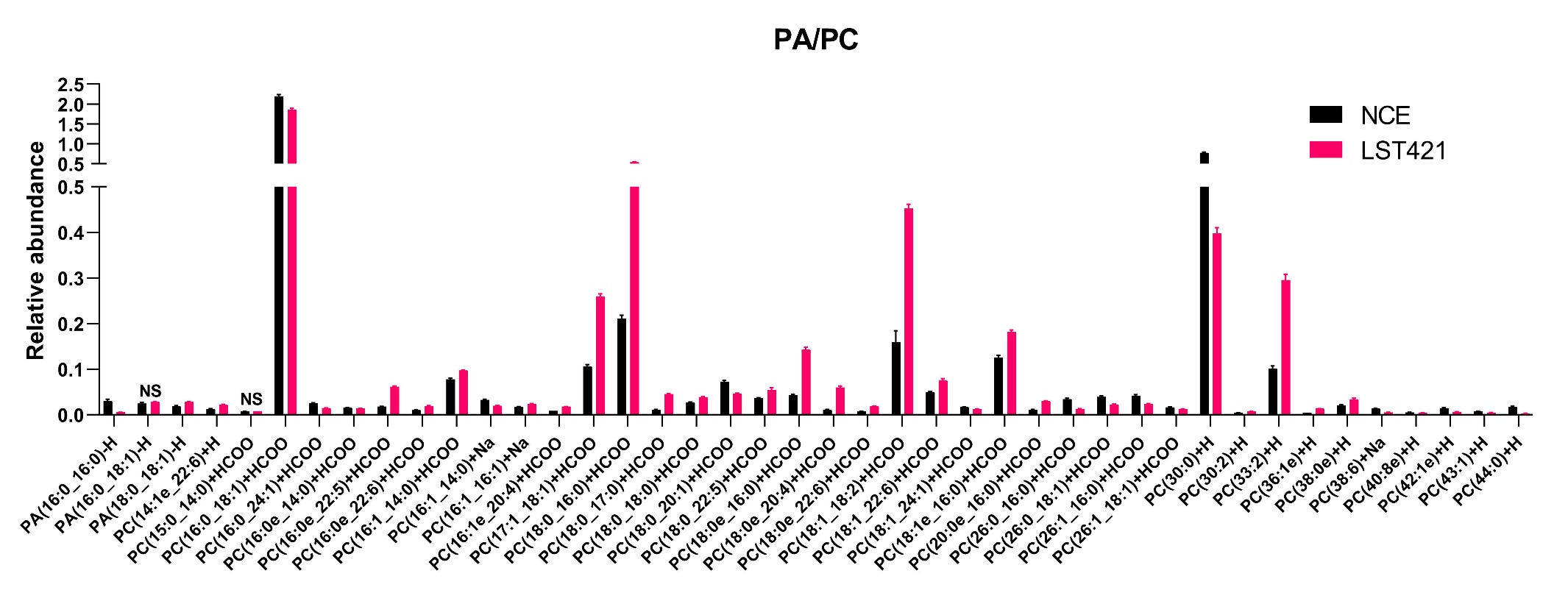
**

**Figure S6**

**
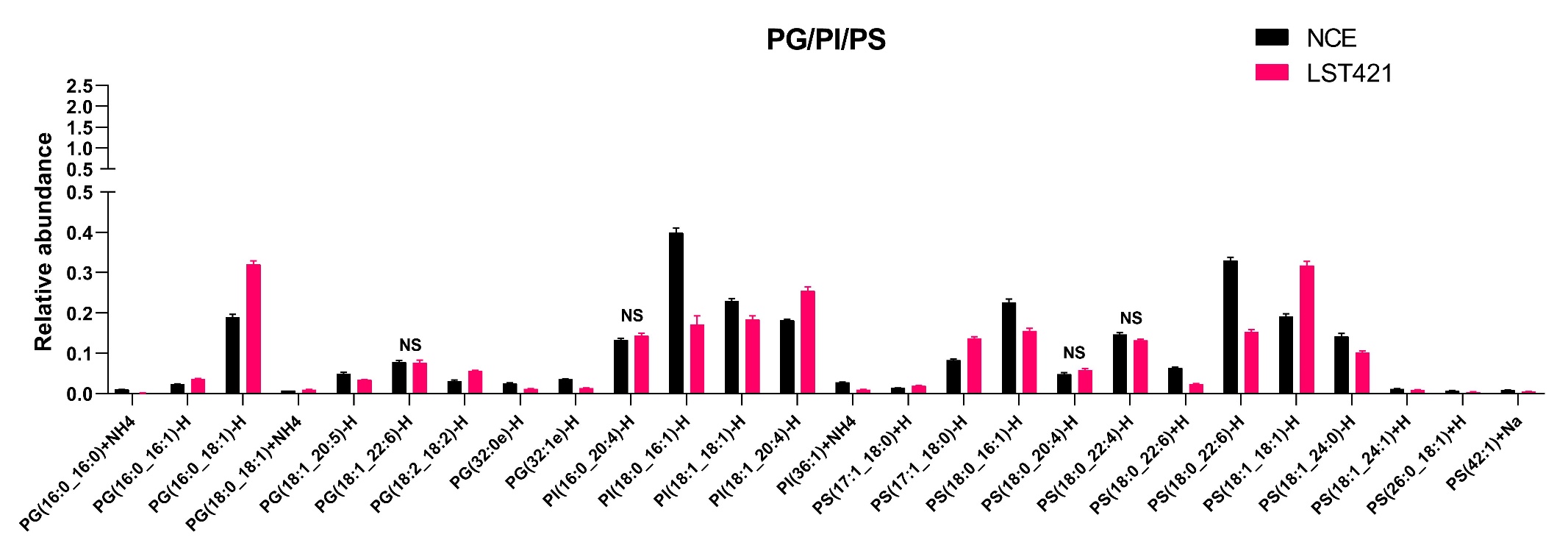
**

**Figure S7**

**
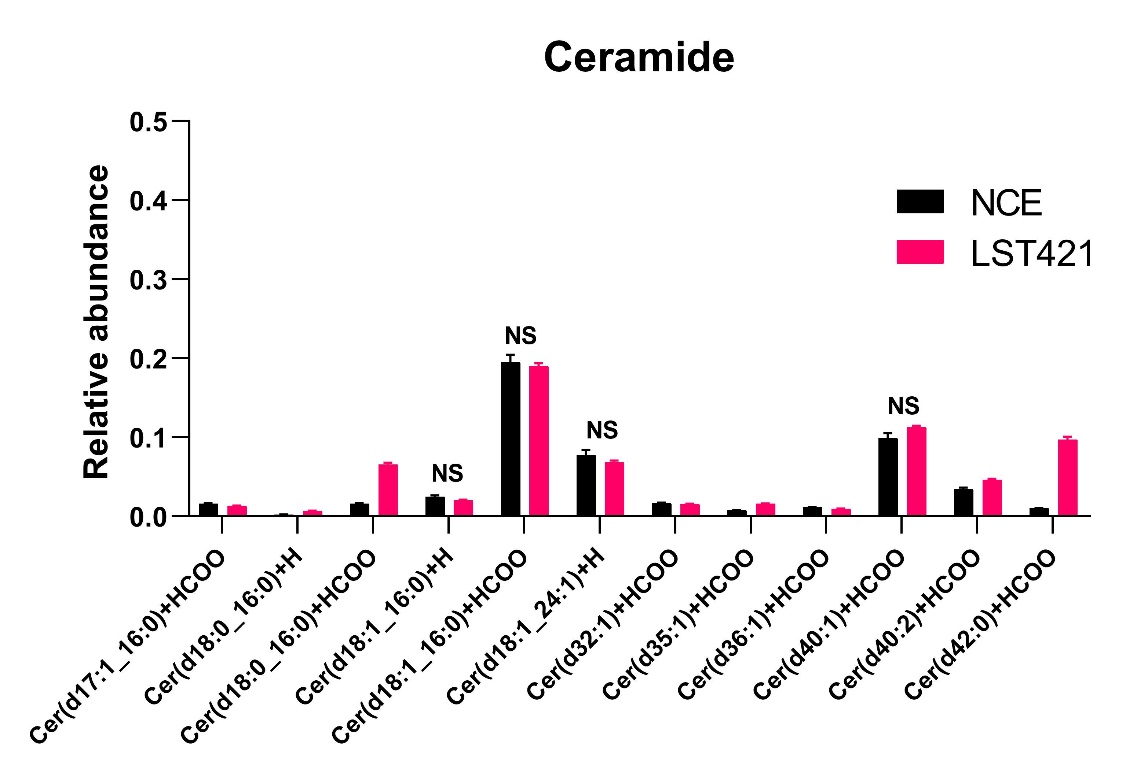
**

**Figure S8**

**
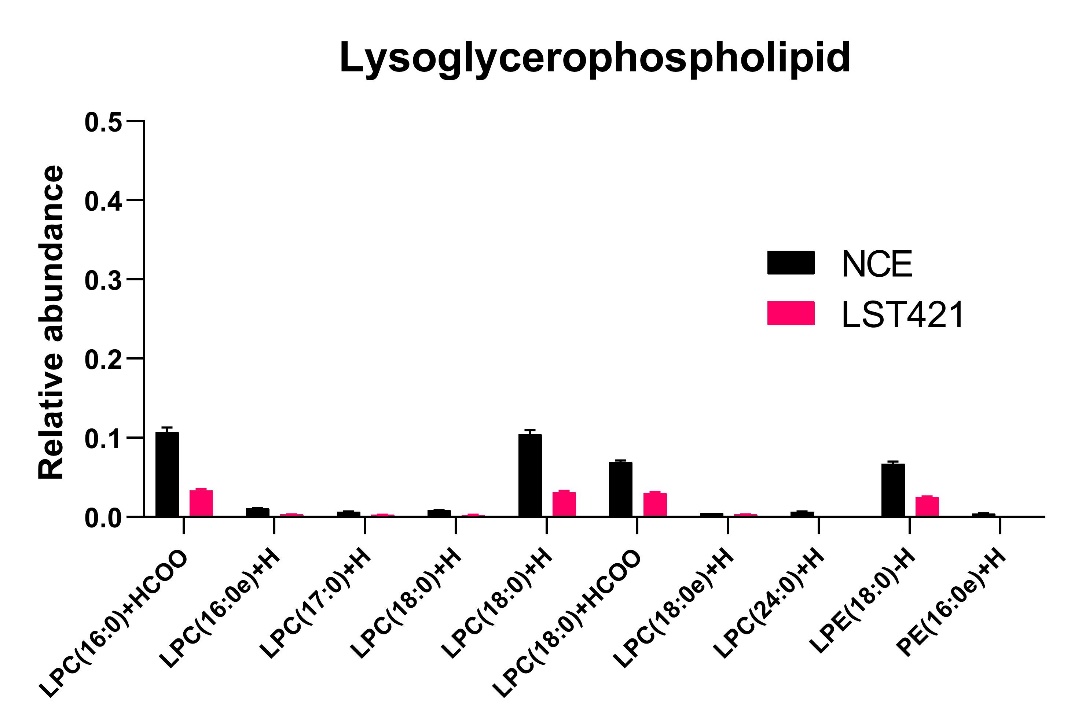
**

**Figure S9**

**
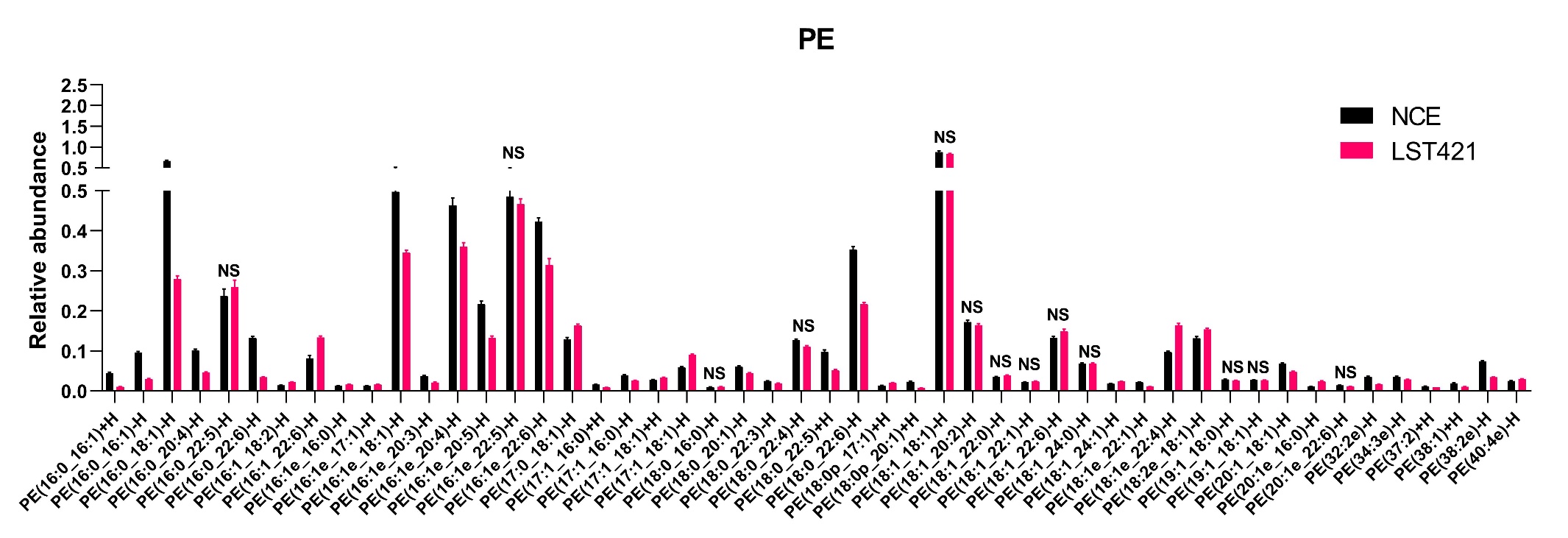
**

**Figure S10**

**
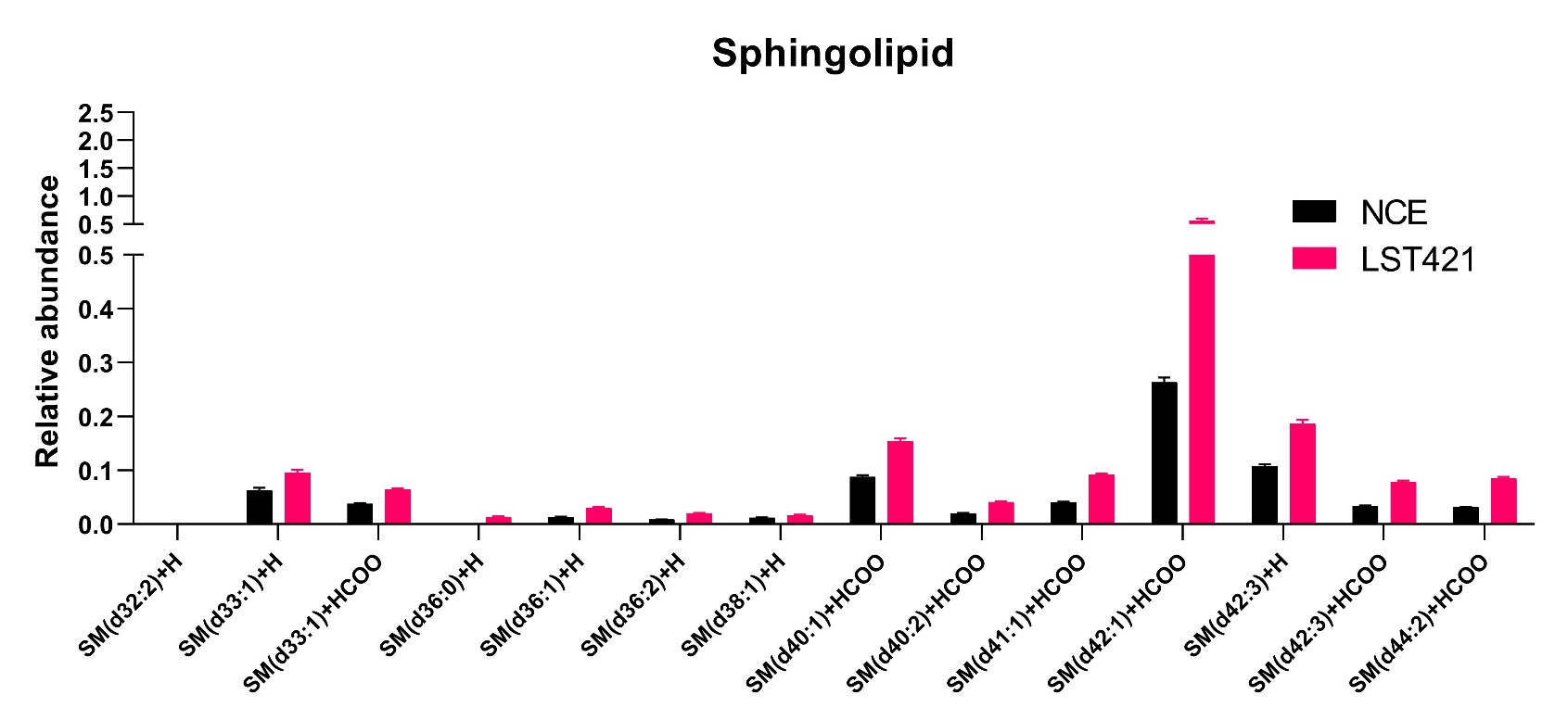
**

**Figure S11**

**A**

**
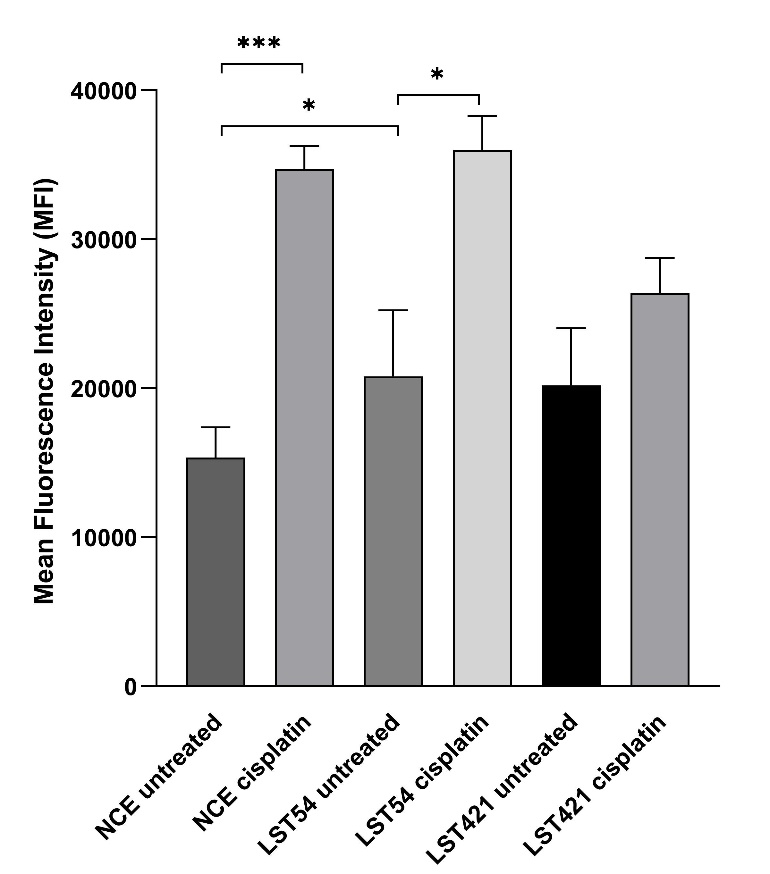
**

**B**


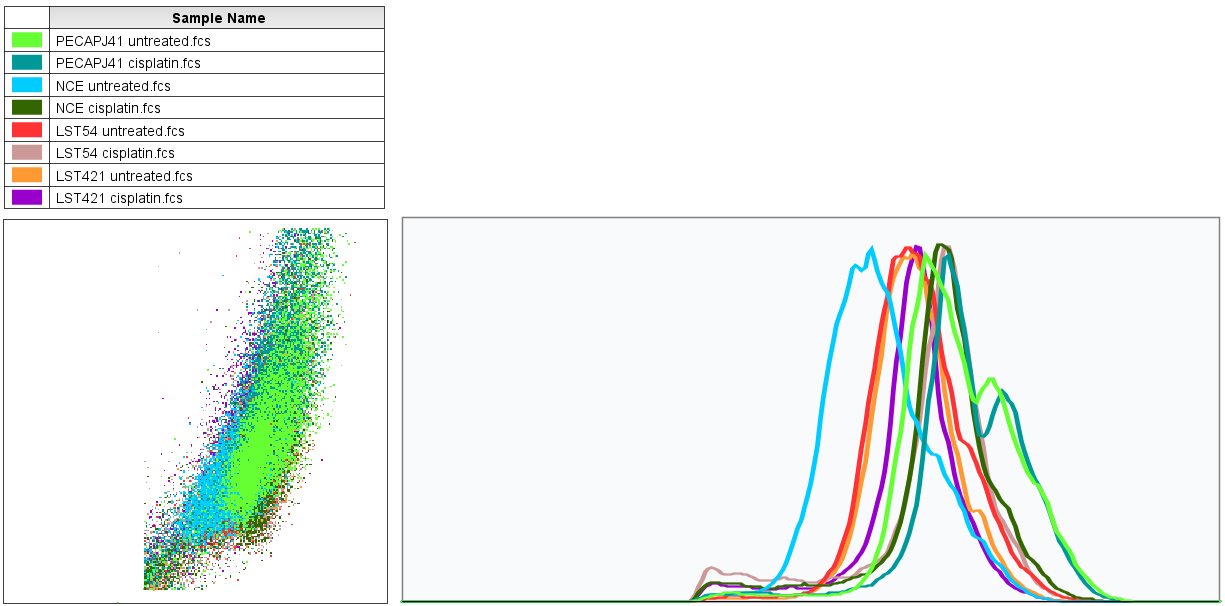


**Figure S12**

**Table S1:** Differentially regulated metabolites in cytoglobin (LST421) expressing cells compared to un-transfected control cells, which were confirmed with MS/MS spectra. A complete list of significantly altered metabolites shown in Table S2.

| **Metabolite class** | **Metabolites up-regulated** | **Metabolites down-regulated** |
| --- | --- | --- |
| Phosphatidic acid | 2 | 1 |
| Phosphatidylcholine | 27 | 16 |
| Phosphatidylglycerol | 4 | 5 |
| Phosphatidylinositol | 2 | 3 |
| Phosphatidylserine | 3 | 8 |
| Ceramides | 6 | 6 |
| Lysoglycerophospholipid | 0 | 10 |
| Phosphatidylethanolamine | 19 | 31 |
| Sphingomyelin | 14 | 0 |
| Cardiolipin | 24 | 1 |

**Table S2**: Spreadsheet containing putative and confirmed MS/MS ion spectra identifications along with associated relative fold change of experimental conditions (NCE, cytoglobin expressing LST 421, NCE cells treated with cisplatin and cytoglobin expressing LST421 cells treated with cisplatin) and statistical significance testing.

Active link to the Microsoft xcel file included in submission named data for Table S2
